# Accessible Eco-Friendly Method for Wastewater Removal of the Azo Dye Reactive Black 5 by Reusable Protonated Chitosan-Deep Eutectic Solvent Beads

**DOI:** 10.3390/molecules29071610

**Published:** 2024-04-03

**Authors:** Oscar Martínez-Rico, Lucía Blanco, Ángeles Domínguez, Begoña González

**Affiliations:** Chemical Engineering Department, Universidade de Vigo, 36310 Vigo, Spain; oscar.martinez.rico@uvigo.es (O.M.-R.); slblanco@uvigo.es (L.B.); admguez@uvigo.es (Á.D.)

**Keywords:** textile wastewater remediation, adsorption, reactive black 5, azo dye, dye removal, chitosan, protonation, deep eutectic solvent

## Abstract

A novel approach to enhance the utilization of low-cost and sustainable chitosan for wastewater remediation is presented in this investigation. The study centers around the modification of chitosan beads using a deep eutectic solvent composed of choline chloride and urea at a molar ratio of 1:2, followed by treatment with sulfuric acid using an impregnation accessible methodology. The effectiveness of the modified chitosan beads as an adsorbent was evaluated by studying the removal of the azo dye Reactive Black 5 (RB5) from aqueous solutions. Remarkably, the modified chitosan beads demonstrated a substantial increase in adsorption efficiency, achieving excellent removal of RB5 within the concentration range of 25–250 mg/L, ultimately leading to complete elimination. Several key parameters influencing the adsorption process were investigated, including initial RB5 concentration, adsorbent dosage, contact time, temperature, and pH. Quantitative analysis revealed that the pseudo-second-order kinetic model provided the best fit for the experimental data at lower dye concentrations, while the intraparticle diffusion model showed superior performance at higher RB5 concentration ranges (150–250 mg/L). The experimental data were successfully explained by the Langmuir isotherm model, and the maximum adsorption capacities were found to be 116.78 mg/g at 298 K and 379.90 mg/g at 318 K. Desorption studies demonstrated that approximately 41.7% of the dye could be successfully desorbed in a single cycle. Moreover, the regenerated adsorbent exhibited highly efficient RB5 removal (80.0–87.6%) for at least five consecutive uses. The outstanding adsorption properties of the modified chitosan beads can be attributed to the increased porosity, surface area, and swelling behavior resulting from the acidic treatment in combination with the DES modification. These findings establish the modified chitosan beads as a stable, versatile, and reusable eco-friendly adsorbent with high potential for industrial implementation.

## 1. Introduction

Textile industries are among the primary pollution generators: the use of large amounts of water and synthetic chemicals generates high volumes of colored wastewater that are usually discharged into aquatic ecosystems. This poses a serious concern as light penetration is hindered, which affects photosynthetic activity in aquatic organisms [1]. Approximately 20% of water pollution on a global scale is attributed to the dyeing and finishing processes involved in textile production [2]. Hence, the development and improvement of techniques for the adequate treatment of textile wastewaters are an urgent need. Textile dyes can be categorized according to their molecular structures. Azo dyes are the largest group of synthetic dyes, and because of their versatility, they account for about 80% of total dyes used in the textile sector [1]. Among the vastly used azo dyes, Reactive Black 5 (RB5) is one of the most consumed reactive dyestuffs in terms of volume. Azo dyes are mainly applied in dyeing of cotton but also viscose, silk, wool, and synthetic fibers [3].

A wide variety of methods have been applied to deal with dye removal, but many of them are not effective either due to insufficient efficiency or for economic reasons [4,5]. Adsorption processes require a relatively simple operating procedure and have proved adequate in the removal of pollutants such as heavy metals and dyes from effluents [6,7]. There is an increasing interest in the application of cheaper and greener adsorbents. Chitosan, an abundant polymer in nature, has gained relevance lately as it can be used as biosupport to synthesize adsorbent materials. Its chemical structure exhibits an abundance of positively charged amine groups (−NH_2_) and negatively charged hydroxyl groups (−O^−^) distributed along the linear cationic polysaccharide backbone. These molecular features confer a significant number of available active sites, ready for diverse chemical interactions and reactivity. Furthermore, different modifications of its structure to improve its efficiency of adsorption and its recyclability have been successfully performed [8].

In a prior investigation, we employed a novel approach to enhance the adsorption capacity of chitosan beads by introducing modifications with a deep eutectic solvent (DES), specifically choline chloride/urea at a molar ratio 1:2 [9], aiming to modify the chitosan surface by attaching functional groups that act as chelating sites, as is the case of the carbonyls coming from urea. DESs represent a class of chemical compounds formed by the combination of two or more pure-state compounds, one being the hydrogen bond donor (HBD) and the other acting as the acceptor (HBA). Through precise mixing at specific molar ratios, these components engage in hydrogen bonding and van der Waals interactions, ensuing in a reduction in the melting point of the solvent and the generation of a liquid-state solvent in a wide range of temperatures. DESs offer an attractive cost-effective alternative as the substitutes of ionic liquids and organic solvents, since they are simple to synthesize and the majority of them are characterized by their non-toxicity and biodegradability. To date, they have been scarcely used for dye removal [10], and only a few studies have combined DES with polymers to obtain eco-friendly adsorbents [11,12,13,14,15].

In the present study, we explore the possibility of a further improvement of the chitosan-DES-impregnated adsorbents by acidic treatment of the beads, since protonation can improve the adsorption of molecules onto chitosan. Previously, we observed that the chitosan-DES beads exhibited great efficacy in the elimination of the anionic dye Acid Blue 80 even after five successive uses when the adsorbent was subjected to acid treatment and then water-washed, maintaining the same adsorption capacity during the study, with no need to desorb the dye [9]. Protonated chitosan-based adsorbents have been scarcely tested for contaminants, as reported in few publications, most of them on metals and other inorganic contaminants [16,17,18,19,20,21] and very few ones on dyes [12,22,23]. Chitosan applications have been partly limited due to its dissolution in an acid medium. An improvement in stability can be achieved by cross-linking methods, establishing bonds that link one polymer chain to another. However, chemical cross-linking with the usual cross-linking agents, epichlorohydrin and glutaraldehyde, reacts with the hydroxyl and amino groups in chitosan, leading to a diminished adsorption capacity of the polymer. Different strategies for improving the stability of chitosan but also the adsorbing performance have been tested, such as the use of amino-group protective agent, followed by a cross-linking reagent and then removal of the protection and protonation [19]. Another approach was the ionic cross-linking of chitosan beads with sulfuric acid [24]. When the chemically cross-linked chitosan was further protonated, the positive charges increased, and so did the ability to remove anionic dyes from aqueous solutions [23].

The objective in this research was to obtain a powerful adsorbent by application of sulfuric acid to the DES-modified chitosan (Ch-DES) beads, as summarized schematically in Figure S12. This is the first report that treats Ch-DES beads with sulfuric acid using a simple method to achieve a stable, reusable eco-friendly adsorbent. This study describes the preparation of the beads and the characterization and testing of the obtained adsorbents, compared to the non-acid treated chitosan beads and chitosan-DES beads. The most efficient adsorbent was elected for a further characterization of the adsorbing properties towards the dye Reactive Black 5. The experimental investigation studied various parameters, including initial dye concentrations, contact time, and adsorbent dose, to assess their impact of the adsorption process. Furthermore, adsorption kinetics and isotherms were analyzed to gain insights into the adsorption behavior. The recyclability of the adsorbent was explored, and a comprehensive understanding of the underlying adsorption mechanism was achieved.

## 2. Materials and Methods

### 2.1. Chemicals

Table S1 provides an overview of the chemicals employed in this study, with their suppliers, purity, and CAS number. The molecular structures of the reagents and RB5 are depicted in Figure S1. Aqueous solutions were prepared with water of Milli-Q quality (mQ). Chitosan with a molecular weight (MW) ranging from 100,000 to 300,000 was sourced from Acros Organics. The deacetylation degree (DD) of chitosan was determined as 84.8% (DD) using Fourier-transform infrared spectroscopy (FTIR), following the procedure outlined in the Supplementary Materials (Equation (S1)).

### 2.2. Synthesis of the Adsorbents

#### 2.2.1. Unmodified Chitosan Beads

An ionic cross-linking method was carried out to synthesize the unmodified chitosan beads (un-Ch), which involved the interaction between opposite charges, following established methodologies outlined in previous studies [25]. Briefly, chitosan was dissolved in acetic acid 2% (*v*/*v*), in the proportion 40 g/L, mixed for at least 24 h at 333.16 K, until a homogenous gel was formed; then it was dropped into a solution of NaOH (2.5 M), and spheroid beads were produced. Following an overnight immersion in the NaOH solution, the wet beads underwent a meticulous washing process using tap water to ensure that the excess NaOH had been completely removed. Subsequently, the beads were carefully rinsed with water until reaching a pH level between 6 and 7. Ultimately, the beads were dried at room temperature to attain their final dry state.

#### 2.2.2. DES-Modified Chitosan Beads

To prepare the DES, a mixture of choline chloride and urea in a 1:2 molar proportion was combined and heated at 353.16 K under agitation until obtaining a single liquid phase. The resulting solution was left to stand to cool down. The subsequent fabrication of the DES-modified chitosan beads was carried out with the following procedure: the wet beads prepared and washed by the procedure described above were placed on filter paper to remove excess water and were combined immediately with recently prepared DES in a 1:1 weight ratio. They were left under vacuum and stirring at 333.16 K overnight, left to stand for 24 h, and washed and left to dry at room temperature.

#### 2.2.3. Sulfuric Acid Treatment for Unmodified Chitosan (ST-un-Ch) and DES-Modified Chitosan Beads (ST-Ch-DES)

Beads prepared as indicated, either un-Ch or Ch-DES beads, were soaked first in purified water for 5 h under stirring at 298.16 K. Filter paper was used to eliminate excess water, and the beads were introduced in a solution of sulfuric acid 0.1 M for 5 h under stirring at 298.16 K. After that, they were rinsed with abundant water until neutral pH. Lastly, they were left to dry at room temperature.

### 2.3. Characterization

The chemical alterations on the beads before and after the procedures (addition of DES and acid treatment) were tracked by Fourier-transform infrared (FTIR). The infrared spectra were acquired using a Nicolet 6700 instrument (Thermo Scientific) (Waltham, MA, USA) coupled with a diamond crystal ATR accessory (Smart Orbit) (Waltham, MA, USA), employing Attenuated Total Reflection (ATR) Spectroscopy.

Another relevant parameter was the structural change induced by the treatments: the surface structures and morphology of the beads were observed using a JEOL JSM 6700F field emission scanning electron microscope (SEM) (Munich, Germany). The TriStar II Plus 3030 system (Micromeritics, Inc.) (Norcross, GA, USA) was utilized to conduct Brunauer–Emmett–Teller (BET) surface area analysis and Barrett–Joyner–Halenda (BJH) pore size and volume analysis.

Swelling behaviors of the different kinds of beads in water and in sulfuric acid 0.1 M were studied. Aliquots of 0.015 g of beads were immersed into 5 mL of each solvent under constant stirring (350 rpm) at 298.16 K until the swelling equilibrium was reached. The swollen samples were taken at varying times, by separating them and eliminating excess of the solution with filter paper. The swelling ratio S (%) was calculated as described by Equation (1) [26].

(1) $$\;S\;\left(\%\right)=\frac{\left(w_{s}-w_{0}\right)}{w_{0}}\times 100\;\;\;$$

where *w_0_* and *w_s_* are the weights of the dry and swollen samples, respectively. The diameters were also measured, using the software ImageJ 1.53k (version 1.54f) on the images of the swollen beads, obtained from a Bresser LCD Microscope (Rhede, Germany).

### 2.4. Adsorption Assays

Dye stock solutions of concentrations between 25 and 250 mg/L were prepared by weighing directly. Additionally, higher RB5 concentrations, up to 2000 mg/L, were prepared for obtaining the adsorbent saturation. No modification of pH was performed (except for the pH effect study). Batch adsorption experiments were conducted to evaluate the influence of various parameters on the adsorption process. The parameters investigated included adsorbent dosage ranging from 0.005 g to 0.035 g, concentrations between 25 mg/L to 250 mg/L for the initial dye solutions, adsorption times going from 15 to 480 min, pH values within the range of 3.6 to 9, and temperatures of 298.16 K and 318.16 K. The experiments were performed in an Ohaus orbital shaker model ISHD16HDG (Nänikon, Switzerland), operating at a continuous shaking speed of 350 rpm. A monitorization of the pH of the solutions before and after each experiment was carried out with a pH-meter Hannah HI5221 (Bedfordshire, UK). The volume of the solutions used in all experiments was maintained at 5 mL. The concentration of the dyes was determined using a Jasco V-750 UV-VIS spectrometer (Tokyo, Japan) at a wavelength of 597.5 nm. Equations (2)–(4) were employed to calculate the equilibrium dye uptake by the adsorbents (q_e_, mg/g), the dye concentration at time t (q_t_, mg/g), and the percentage of dye removal.

(2) $$q_{e}=\frac{V{(C}_{0}-C_{e})}{m}\;;$$

(3) $$q_{t}=\frac{V{(C}_{0}-C_{t})}{m}\;;\;$$

(4) $$Dye\;removal\;\%=\frac{C_{0}-C_{t}}{C_{0}}\times 100$$

The dye concentrations (mg/L) at the beginning, at time *t* (min), and at equilibrium are *C*_0_, *C_t_*, and *C_e_*, respectively. The mass of the adsorbent (g) is *m*, and the dye volume (L) is denoted as *V*. At least two replicate experiments were performed to calculate the mean result, until a value lower than 10% for the variation coefficient was achieved, unless otherwise specified. The data are presented as mean values ± standard error of the mean (Equation (S2)).

The initial evaluation of the four adsorbents with the dye was performed under the following experimental conditions: 3 g of adsorbent per L of dye solution, 150 mg/L of dye in the initial solutions, 298.16 K, and 7 h of contact time. The adsorption equilibrium and the kinetics of the best tested adsorbent were studied more deeply. Table 1 summarizes the results of the four series of adsorption experiments, with their different sets of conditions.

### 2.5. Adsorption Kinetics

Pseudo-first- and pseudo-second-order kinetic models (Equations (S4) and (S5), respectively), Elovich kinetic model (Equation (S6)), and intraparticle diffusion (Equation (S7)) [27,28,29] were used to analyze the results obtained.

### 2.6. Adsorption Isotherm

Langmuir, Freundlich, Temkin, Elovich, and Dubinin–Radushkevich isotherm models [30,31,32,33,34] were utilized to examine the adsorption mechanism of the dye towards the sorbent, as well as its affinity and trend. Equations (S8)–(S15) represent the mentioned models, respectively.

### 2.7. Desorption and Reusability Tests

To assess the RB5 desorption, a series of basic and acid solutions were used: NaOH at varying concentrations (0.1 M, 1.0 M, 2.5 M), KOH 1.0 M, NH_4_OH, H_2_SO_4_ 0.1 M, and HCl (0.1 M, 0.01 M). Approximately 0.015 g of the beads employed in preceding RB5 adsorption experiments was put in contact with 5 mL of these solutions and shaken for 24 h. Then, the solubility of the beads and the potential for RB5 desorption were observed. The best ones were chosen, and desorption series of varying duration were carried out. Subsequently, an assessment of the adsorbents’ reusability was conducted. In addition, the reusability of the untreated adsorbent was investigated, employing 0.015 g of adsorbent, 5 mL of solution, an initial RB5 concentration of 150 mg/L, a contact time of 420 min, and a temperature of 298.16 K.

The regeneration capability of the adsorbent was also assessed: the beads were firstly used to adsorb RB5, and then, they were treated with NaOH 0.1 M (7 h) to achieve a partial desorption, washed with water, regenerated with H_2_SO_4_ 0.1 M for 5 h, again washed with water, and finally reused to adsorb the dye. Several cycles of desorption, regeneration with 0.1 M sulfuric acid, and reutilization with RB5 dye were performed.

## 3. Results and Discussion

### 3.1. Adsorbent Characterizations

Identification of the possible chemical and physical changes and characterization of the modifications on the beads were carried out in order to understand the complete processes and rationalize the adsorption results. Therefore, the adsorbents were analyzed by FTIR to obtain the spectra of the starting materials and the beads prepared by the different methods, so as to confirm the changes and occurrence of different functional groups. The structural changes induced by the different treatments were also studied by BET surface area analysis and BJH pore size and volume analysis. The SEM images were also useful to visualize the surface structures and morphology of the beads.

#### 3.1.1. Chemical Characterization by Fourier-Transform Infrared (FTIR) Spectroscopy

The structural transformations on the beads carried out with these procedures (impregnation with DES and acid treatment), i.e., the different functional groups, were elucidated by FTIR spectroscopy.

The FTIR spectra of the adsorbents before the acidic treatment and individual components are shown in Figure S2. Table S2 presents the primary functional groups.

Spectral analysis revealed discernible distinctions between Ch-DES beads and un-Ch beads (Figure S2), suggesting potential modifications in the attached functional groups. In the 1500–1700 cm^−1^ region, unmodified chitosan exhibited two characteristic bands associated with the bending of N-H bonds and the stretching vibration of C=O. However, DES-modified chitosan beads displayed the emergence of three bands: a band at 1554 cm^−1^, in addition to the more intense 1600 cm^−1^ band, which could be attributed to the presence of DES. Previous studies have proposed that these bands at 1554 cm^−1^ and 1600 cm^−1^ correspond to the bending of N-H bonds in amide and NH_2_ groups. Furthermore, the band at 1646 cm^−1^, associated with the stretching vibration of C=O in the amide I group of chitosan beads, somewhat shifted to 1650 cm^−1^ in chitosan-DES beads. In comparison to unmodified chitosan beads, Ch-DES exhibited a noticeable increase in the area under this peak. This observation suggests the possible contribution of other C=O urea bands originating from the DES, confirming the attachment of DES molecules to the chitosan beads. The intensity variations in these bands suggest the presence of more N-H and C=O groups resulting from the urea in DES, as previously reported by our research group [9].

**Figure 1:**
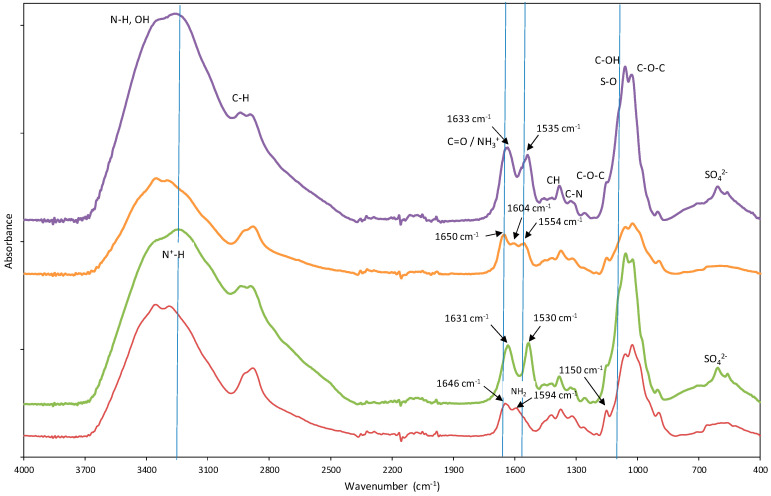
Fourier-transform infrared (FTIR) spectra of the chitosan-based adsorbents, highlighting the spectral differences among them: unmodified chitosan (**─** un-Ch) beads, acid treated unmodified chitosan (**─** ST-un-Ch) beads, DES-modified chitosan (**─** Ch-DES) beads, and acid treated modified chitosan (**─** ST-Ch-DES).

The ST-Ch-DES beads and ST-un-Ch beads show clear differences with Ch-DES and un-Ch beads, respectively (Figure 1). The band at 1650 cm^−1^ in the chitosan-DES beads (stretching vibration of C=O of amide I group) slightly shifts to a lower wavenumber: 1633 cm^−1^ in the acid-treated Ch-DES beads. Analogous observations can be performed in the un-Ch beads. The band at 1554 cm^−1^ in chitosan-DES beads shifts to a lower wavenumber: 1535 cm^−1^. These bands are possibly contributed by the amine functional group −NH_3_^+^, which shows absorptions near 1600 and 1500 cm^−1^ due to asymmetric and symmetric deformation vibrations [35]. Similar findings were described when sulfuric acid was used in chitosan membranes, and new absorption bands characteristic of NH_3_^+^ bending appeared at 1634 and 1531 cm^−1^, while the band at 1587 cm^−1^ disappeared, suggesting that the NH_2_ chitosan groups became protonated [36]. Other important changes are as follows: the small band around 1150 cm^−1^ in both adsorbents before acid treatment, typical of C-O-C stretching vibration, became less evident by the occurrence of a broader band in the region around 1090 cm^−1^, possibly explained by S-O stretching vibrations, and another band appeared at 606 cm^−1^, which can be attributed to the presence of SO_4_^2−^ ions, also described in similar chitosan modification experiments [36].

Regarding the spectral range 4000–2000 cm^−1^, there were also differences between the acid-treated adsorbent and the Ch-DES beads: the band at 3340 cm^−1^ in the untreated adsorbents is not so evident in the acid treated adsorbents, and the bands became broader, and rounded near 3200 cm^−1^, this absorption can be assigned to the stretching vibration of N^+^-H [37], showing that the NH_2_ groups in chitosan were protonated to NH_3_^+^ [24]. This broad band was described to be sensitive to SO_4_^2−^ ions, because of the strong interaction between the SO_4_^2−^ and NH_3_^+^ ions, and the more SO_4_^2−^ ions interacted with the NH_3_^+^ groups, the broader the band at 3200 cm^−1^ [36]. There were also changes in the C-H stretching band: the band shifted to higher wavenumbers (range 2892–2951 cm^−1^). It has been reported that when glucosamine units in chitosan are protonated, hydrogen bonding involving the NH_2_ groups is disrupted, and the rigid crystalline structure weakens; on the other hand, the infrared C-H stretching bands shift to lower wavenumbers and become sharper as crystallinity increases [36].

This FTIR spectra suggested that un-Ch and Ch-DES beads treated with H_2_O-H_2_SO_4_ were modified by H_2_SO_4_ through the ionic interaction between the protonated amine groups (−NH_3_^+^) of chitosan and the SO_4_^2−^ ions of sulfuric acid as also described by others [24].

#### 3.1.2. Morphological Study by Scanning Electron Microscopy (SEM)

**Figure 2:**
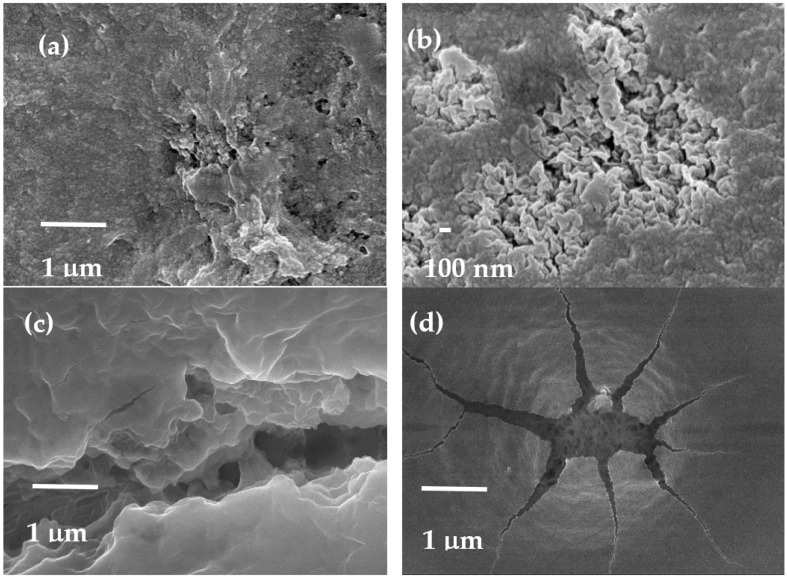
SEM photographs of the adsorbents (**a**) unmodified chitosan (un-Ch), (**b**) chitosan-DES (Ch-DES), (**c**) sulfuric-acid-treated unmodified chitosan (ST-un-Ch), and (**d**) sulfuric-acid-treated chitosan-DES (ST-Ch-DES) beads.

Figure 2 illustrates the morphological characteristics of the un-Ch, Ch-DES beads, as well as the treated ones (ST-un-Ch and ST-Ch-DES), as observed through scanning electron microscopy. In all cases, the beads displayed a spherical-ovoid shape (Figure S3) with an average diameter of approximately 1 mm. Both the unmodified adsorbents, un-Ch and Ch-DES, exhibited smooth surfaces with minor surface cavities. Notably, Ch-DES demonstrated regions with a rougher surface, showcasing a higher occurrence of cavities and small pores (Figure 2a,b). By contrast, sulfuric-treated beads exhibited deeper cavities with higher pore volume (Figure 2c,d), with ST-Ch-DES showing a more irregular surface: although most of the surface was regular and smooth, it exhibited cracks with openings leading to an inner porous space. In addition, ST-Ch-DES showed greater variability, with beads exhibiting altered shapes, fractured and opened, among others with a more regular spheroid shape (Figure S3).

#### 3.1.3. Brunauer–Emmett–Teller (BET) and Barrett–Joyner–Halenda (BJH) Analysis

Brunauer–Emmett–Teller (BET) and Barrett–Joyner–Halenda (BJH) analysis was performed in order to determine the surface area, volume, and pore size of the beads. Results are shown in Table 2.

The small pore size of all the beads was comparable to the values for chitosan beads reported in the literature [38] or slightly higher [39]. However, the pore volume and the BET surface area were notably increased in the ST chitosan beads: six-fold in the ST-un-Ch beads and 12-fold in the ST-Ch-DES beads, taking the corresponding untreated beads as reference, respectively. The same observation can be made for the micropore surface area. We can conclude that while in the original adsorbents, the porosity was very low, it has significantly increased in the acid-treated ones. Similar increases in BET surface area and pore volume were reported in chitosan treated with phosphoric acid [40]. Other authors also reported that the surface of the chitosan beads was less porous before the acidic treatment and became more porous and coarser after the acid treatment, which induced an ionic cross-linking process [24]. 

#### 3.1.4. Swelling Behavior

Previous studies pointed out the direct correlation between the swelling behavior of the beads and their ability to incorporate chemicals from their surroundings [41]. In addition, the increase in surface area is generally beneficial for adsorption. However, the increase in swelling means a higher amount of water molecules entering the beads, which could decrease the adsorbent stability. Then, to characterize the adsorbents, the swelling of the Un-Ch and Ch-DES beads was investigated as a function of time in two different solvents: water (W) and 0.1 M sulfuric acid (A), separately, and additionally, in water until equilibrium was reached and followed by 0.1 M sulfuric acid (W-A). Table 3 shows the obtained values: weight increase, expressed as swelling ratio, and the diameter of the beads after reaching equilibrium. Ten individual beads taken from each of at least three samples were measured to quantify the diameter increase. 

All the beads increased their weight and size, and the weight rose particularly when acid was used, in both adsorbents. Combined treatment of water followed by sulfuric acid produced a remarkable swelling ratio in the Ch-DES beads (about 226%) compared to un-Ch beads (119%) and a higher diameter increase (44% vs. 30%). The obtained results showing that acidic medium caused a higher swelling (Table 3) might be explained by the ionization: chitosan amino and imine groups became protonated as the acid solvent penetrated from the surface to the core of the beads. Hydrogen bonds among the amino groups weakened, and the osmotic pressure inside the beads induced a faster uptake from the medium [41]. The higher swelling of Ch-DES beads versus un-Ch both in water and in acid would be explained by a lower cross-linking intensity, since DES acts as a plasticizer and leads to separation of polymer strands, with a decrease in the strength of the intermolecular interactions. A higher hydrophilic character and the more flexible Ch-DES beads would also explain this behavior: choline-chloride-based-DES was reported to enhance the elasticity of chitosan [37], in contrast to the more brittle un-Ch beads. This could also be the explanation for the highest swelling observed on the Ch-DES beads after the combined treatment of water followed by sulfuric acid: beads previously soaked in water propitiated acid flow, and the higher number of attached N-H groups coming from the DES in the Ch-DES beads promoted and multiplied the ionization degree and, accordingly, the swelling rate. Therefore, the Ch-DES beads offered the most interesting swelling properties, especially when they were treated with water and 0.1 M sulfuric acid afterwards (ST-Ch-DES).

### 3.2. Selection of the Best RB5 Adsorbent

Both FTIR and BET analyses confirmed the chemical modifications on the beads produced by the treatments (impregnation with DES and acid treatment) and the structural changes induced by the acid treatment: the protonation of amine groups, their ionic interaction with the SO_4_^2−^ ions of sulfuric acid, and the higher porosity and surface roughness. Based on all these findings, together with the observations on swelling behavior, the adsorption towards the dye RB5 was tested for the ST beads (ST-Un-Ch and ST-Ch-DES) and the original (Un-Ch and Ch-DES) beads as a control, to select the best.

**Figure 3:**
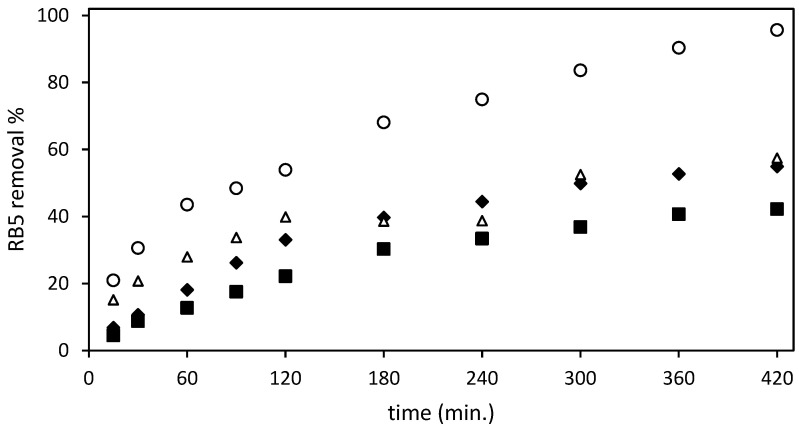
Effect of the different adsorbents and contact time on the dye removal from the aqueous solution (RB5 removal %): (■) un-Ch, (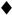) Ch-DES, (∆) ST-un-Ch, (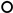) ST-Ch-DES; at 298.16 K with a ratio adsorbent mass/dye solution volume of 3 g/L.

Figure 3 shows the RB5 removal data obtained after testing the four different adsorbents (un-Ch, Ch-DES, ST-un-Ch, and ST-Ch-DES) at an initial RB5 concentration of 150 mg/L and adsorbent dose of 3 g/L.

The ST-Ch-DES beads adsorbed the dye RB5 at a higher rate than the remaining tested beads: a sharper increase in the adsorption during the first 60 min. of the experiment was exhibited, compared to the smoother adsorption increase shown by ST-un-Ch and Ch-DES, with un-Ch showing the lower adsorption rate at all intervals of the experiment. Then, in the next interval, from 60 to 300 min., the increase in adsorption was smoother in all the adsorbents tested, except in the ST-Un-Ch, where in the 3 to 4 h range experimental error may be responsible for values of adsorption lower than expected. ST-Ch-DES was the one that removed the dye at a higher rate, reaching nearly 84% at 300 min, with 42.7 mg/g adsorption. Finally, the maximum RB5 removal values, >95%, were reached, and around 50 mg/g of colorant were adsorbed by ST-Ch-DES at 420 min. Within the next hour, until 480 min., absence of color in the solution was achieved. The variation coefficient among replicates was below 19% in all cases, when ST-Ch-DES adsorbent was essayed; on the contrary, ST-un-Ch and non-ST adsorbents replicates were below 10% variation.

Both ST-un-Ch and Ch-DES exhibited comparable adsorption rates, although ST-un-Ch demonstrated a slightly faster initial adsorption. During the next interval, from 120 min. to the end of the test, both adsorbents followed a similar removal pattern, reaching maximum capacity and attaining equilibrium states at approximately 300–360 min. The removal rate during this period ranged from 50% to 57%, with adsorption capacities of around 28.2 mg/g in both adsorbents. Un-Ch shows the lowest rate and reaches the lower adsorption values, around 21 mg/g, and eliminates about 40% of RB5 from the water solution.

Clearly, the ST-Ch-DES beads showed a higher adsorption capacity (Figure 3) when used with the azo dye RB5. Therefore, they were chosen for a more in-depth analysis of their adsorption equilibrium and kinetics.

### 3.3. Variables Impacting the Adsorption Process

#### 3.3.1. Effect of Adsorbent Dose

**Figure 4:**
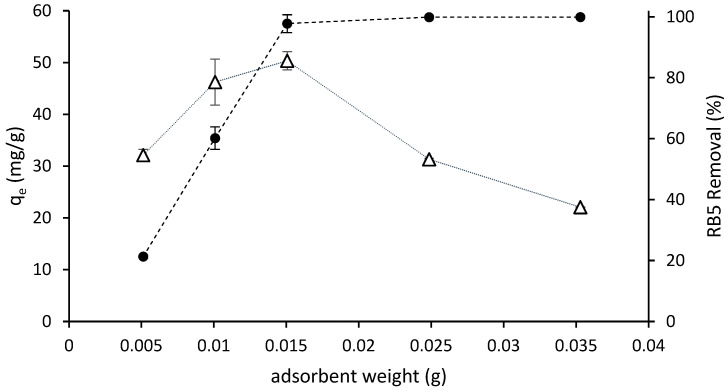
Removal efficiency (●) and amount of dye adsorbed ((q_e_) (∆) as a function of ST-Ch-DES dosage, for 6 h at 298 K from 5 mL of RB5 solution of an initial concentration of 150 mg/L.

The removal efficiency and adsorption of RB5 in aqueous solution as a function of the adsorbent dosage is shown in Figure 4. The removal rate increased at increasing doses of adsorbent and reached the highest values, nearly 100% (practical color absence, see Figure 3), at the 0.015 g dose and higher. The adsorption capacity increased when the adsorbent dose increased from 0.005 to 0.015 g; then it reached the maximum value and decreased at higher adsorbent quantities. The availability of binding sites for adsorption increased when the quantity of adsorbent increased and a higher surface area was available. 

Optimal performance with minimal adsorbent usage can be achieved by employing a dosage of 0.015 g per 5 mL of RB5 solution with an initial concentration of 150 mg/L. This dosage results in the highest adsorption capacity while achieving maximum removal efficiency and reaching the equilibrium state.

#### 3.3.2. Effect of Initial Colorant Concentration and Contact Time

**Figure 5:**
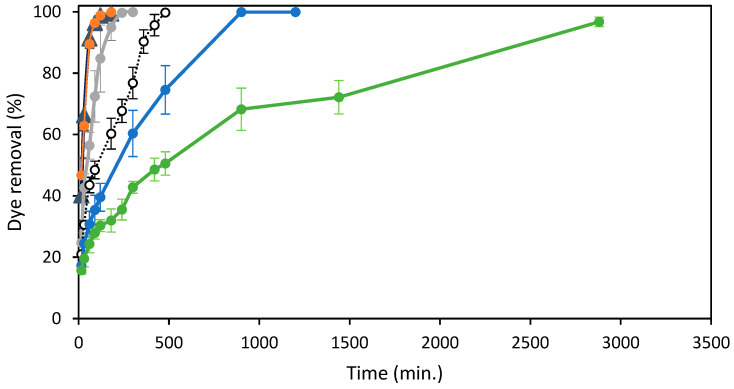
Effect of Reactive Black 5 concentrations and agitation time on the elimination (%) of the dye at 298 K by 15 mg of ST-Ch-DES beads: (▲) 25 mg/L, (●) 50 mg/L, (●) 100 mg/L, (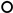) 150 mg/L, (●) 250 mg/L.

Figure 5 shows the effect of adsorption time and initial concentrations of the azo dye RB5 on the removal efficiency of the adsorbent ST-Ch-DES.

As shown, the adsorption was very fast during the first step of the experiment, especially at the lowest colorant RB5 concentrations. At the highest RB5 concentrations, the fast adsorption step was shorter and led to a gradual adsorption increase: the more concentration of dye in solution, the lower the adsorption rate. The dye removal achieved the maximum efficiency, near 100%, at all the RB5 concentrations, including the highest concentration, 250 mg/L, tested in this study, achieving > 96% removal at 48 h. At the lowest RB5 initial concentrations, 25 and 50 mg/L, the removal increased sharply during the first 60 min. and slowed down the next 60 min., until practically total removal at 120 min. When the initial RB5 concentration was 100 mg/L, the removal was not so fast, but it was still high during the first 120 min., with a lower slope compared to the former concentrations and reached the maximum removal values at 240 min. More time was necessary when dealing with more concentrated dye solutions: about 8 h to reach the maximum efficiency near 100% at 150 mg/L, and about 15 h to completely remove the dye at 200 mg/L initial concentration. A removal rate around 68% was reached after 15 h at 250 mg/L initial dye concentration and above 96% at 48 h.

The abundant presence of active adsorption sites on the surface of the beads can account for the initial rapid removal rate observed. During the initial stage, the diffusion of dye molecules from the solution onto the adsorbent surface relies on factors such as dye solution concentration, agitation, and contact time [42]. As the concentration gradient between the adsorbate and adsorbent increases, mass transfer resistance between the two can arise, resulting in reduced removal efficiency at higher initial dye concentrations. Furthermore, as the adsorbent sites get occupied by the adsorbate and the contact time between the dye and adsorbent is prolonged, a surge in the repulsion forces between the adsorbed and the free dye may take place [27].

The amount of dye adsorbed increased rapidly at the initial step, faster and reaching higher values at higher RB5 concentrations, up to 120 min. (Figure S4). From that moment, the adsorption follows a similar pattern until the min. 300 in all the solutions at the highest initial concentrations (100–250 mg/L), when the adsorption is in the range of 34–40 mg/g for all of them. Thereafter, the adsorption increase is the slowest at the highest RB5 initial concentration, while it reaches the maximum value at 100 mg/L, and continues rising at a similar rate at the concentrations of 150 and 200 mg/L. The increasing concentration gradient at higher initial dye concentrations would balance the mass transfer resistance between the adsorbate and adsorbent, explaining these results [27]. The adsorption capacities obtained were 8.6, 16.9, 34.7, 50.8, 69.5, and 83.35 mg/g for RB5 concentrations of 25, 50, 100, 150, 200, and 250 mg/L, respectively, at 298.16 K and pH 6.0 (unmodified pH of RB5 solution in water).

#### 3.3.3. Effect of Temperature

RB5 adsorption experiments were carried out at 298.16 and 318.16 K, in order to test the influence of the temperature on the process. Adsorption capacity remains essentially constant at all temperatures, with dye removal rates around 99% at all the tested temperatures and RB5 initial concentrations of 25–250 mg/L. A sharp increase in adsorption, about 46.5%, can be seen when the temperature goes from 298.16 to 318.16 K at the maximum RB5 concentration, 500 mg/L (Figure S5). This increase in dye removal with temperature would indicate that the adsorption process is endothermic at high RB5 concentrations. 

#### 3.3.4. Effect of pH 

The adsorption behavior at different pHs is an important factor to be considered. The influence of pH on the dissociation or ionization of dye molecules and on the functional groups in the adsorbent should be considered to understand the process, as reported by Patiño-Ruiz et al. [25].

Figure S6 demonstrates the impact of initial pH values on the removal of RB5 using ST-Ch-DES beads. The pH influence was assessed within a range of 3.6 to 9.2, with RB5 initially present at a concentration of 150 mg/L and the removal process monitored over a duration of 480 min. The results revealed that the removal percentage was notably higher at lower pH values. Specifically, at pH 3.6, a removal efficiency of 99.9% was achieved, while at the highest pH tested (pH 9.2), the removal efficiency dropped to 51.4%. Intermediate pH values, approximately 5, 6, and 8, corresponded to removal efficiencies exceeding 96.5%.

Hence, it can be inferred that the adsorption of RB5 onto the ST-Ch-DES beads was not heavily influenced by pH within the range of 3.6 to 8. However, beyond this range, a sharp decline in efficiency was observed. Under acidic conditions, the amine groups of the adsorbent became protonated, facilitating the electrostatic attraction between the negatively charged sulfonate ions of RB5 and the adsorbent surface, consequently accounting for the highest removal percentage at pH 3.6 [25]. As pH increased, the abundance of protonated amino groups on the adsorbent decreased, causing a weakening of the electrostatic interaction between the dye molecules and the ST-Ch-DES beads. For pH values higher than 8, the adsorption capacity experienced a steeper decline, likely due to increased repulsive forces between the anionic dye and the adsorbent surface.

The ST-Ch-DES beads were still able to remove half the amount of a highly concentrated RB5 (150 mg/L) solution at pH 9 (480 min, 298.16 K), within the pH range reported for the textile industry wastewaters (from 9 to 11) [43].

### 3.4. Adsorption Kinetic Models

**Figure 6:**
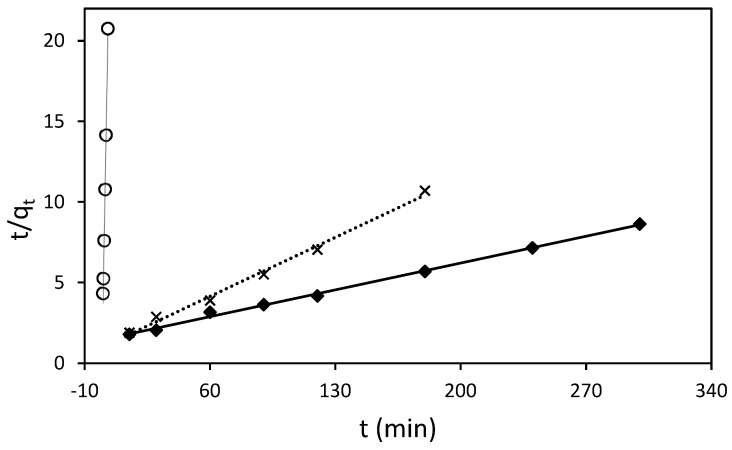
Pseudo-second-order sorption kinetics of RB5 at 298.16 K by 15 mg of ST-Ch-DES beads, at different RB5 initial concentrations: (○) 25 mg/L, (x) 50 mg/L, (♦) 100 mg/L.

**Figure 7:**
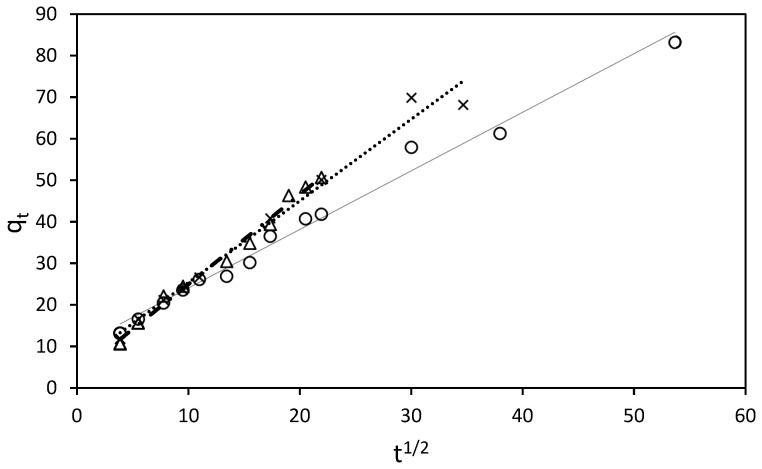
Intraparticle diffusion model for sorption kinetics of RB5 at 298.16 K by 15 mg of ST-Ch-DES beads, at different RB5 initial concentrations: (∆) 150 mg/L. (x) 200 mg/L. (○) 250 mg/L.

The adsorption process of RB5 onto ST-Ch-DES beads at various initial concentrations was investigated to gain insight into the underlying mechanisms and determine the adsorption kinetic model that best describes the process. The pseudo-second-order model (Figure 6), along with the pseudo-first-order, Elovich, and intraparticle diffusion (Weber–Morris model) kinetic models (Figure 7), were employed and represented through linear graphs. The linear plots of ln *(q_e_*-*q_t_)* versus *t*, *t*/*q_t_* versus *t*, *q_t_* vs. *ln*(*t*), and *q_t_* vs. *t*^1/2^, respectively, were utilized to derive the corresponding parameter values. The calculated adsorption kinetic parameters (*k*_1_, *k*_2_, *k*_3_, *q_e_*, *I*, *β*, and *α*), obtained from the linear plots, are presented in Table 4 along with their *R^2^* values.

For the lowest RB5 concentrations (25, 50, and 100 mg/L), the pseudo-second-order model was found to be the most appropriate based on the linear fit and regression coefficient values (Figure 6). The calculated *q_e_* values obtained from this model were in good agreement with the experimental ones. However, it is important to note that the model yielded slightly higher *q_e_* values, ranging from 10% to 23% higher, as indicated in Table 4. The rate constant *k*_2_ calculated decreased when the initial RB5 concentration increased, meaning that the adsorption rate was slower with higher dye initial concentrations, in agreement with the results obtained in the present study (Section 3.3.2). Other authors described data adjustment to the pseudo-second-order model when using protonated adsorbents [23,24].

At higher RB5 concentrations, a better fit between the experimental data and the intraparticle diffusion model (Weber–Morris) was obtained (Figure 7).

The intraparticle diffusion model aids in understanding the distinct adsorption behavior of RB5 at low and high concentration ranges. At low concentrations (25–100 mg/L), the adsorption process can be divided into three steps (Figure S7a–c), each representing different mechanisms of mass transfer. Initially, a film is rapidly formed on the adsorbent surface through external mass transfer, facilitating fast diffusion. Subsequently, a slightly slower adsorption phase is observed, where intraparticle diffusion may become the rate-controlling step. Finally, in the third step, adsorption reaches a state of equilibrium as most of the dye molecules in the solution are already adsorbed. At this stage, intraparticle diffusion slows down due to the extremely low solute concentration remaining in the solution [44]. The rate of RB5 adsorption onto ST-Ch-DES beads depends on the number of active sites and the concentration of the dye in the solution, in accordance with the assumptions of the pseudo-second-order model, which indicates that surface adsorption is the dominant process [5]. This process is governed by chemical adsorption and influenced by electrostatic forces [25].

Conversely, at higher RB5 concentrations (150–250 mg/L), the intraparticle diffusion model provides a better explanation for the adsorption process. Similarly, this process can be divided into three stages (Figure S8): Initially, there is rapid diffusion on the surface, accompanied by the formation of a film on the adsorbent, although this stage is short-lived as a significant number of dye molecules in the solution promptly adhere to the surface. Subsequently, a gradual adsorption step occurs, characterized by slow particle diffusion between the RB5 films formed during the initial stage, as well as interaction with the porous structure within the core of the beads. Finally, in the third stage, the adsorption rate stabilizes as practically total adsorption of the dye molecules in the solution is achieved. After this, extremely low solute concentration in the solutions was observed: in the 150 mg/L dye solution within the first 8 h, in the 200 mg/L solutions, within the first 16 h, and even in the 250 mg/L solution, in a wider time range, 48 h, at 298.16 K. At higher temperatures, the process reached total decolorization at the concentration range 25–250 mg/L in a significant shorter time; at 308 K, all the solutions achieved the equilibrium within the first 5 h except 250 mg/L, reaching total adsorption in less than 24 h, and at 318 K, all the solutions including 250 mg/L, within the first 5 h. Saturation of the active sites or lack of adsorption owing to the absence of available active sites was not detected in our study at the mentioned concentration range (25–250 mg/L). In addition, if the regression curve obtained with the *q_t_* versus *t*^1/2^ plot, according to the Weber and Morris model, passed through the origin, the intraparticle diffusion would be the sole rate-limiting step [44]. Nevertheless, this was not our case (Figure 7), so this would be indicative of some degree of boundary layer control, and some other processes might be controlling the rate of adsorption, besides the intraparticle diffusion [45].

The surface diffusion of the dye molecules to the interior of the adsorbent through the pores seems to be the main mechanism occurring in the ST-Ch-DES beads interaction with the dye RB5, at high initial dye concentrations. This would explain the longer time required to adsorb greater colorant quantities and the higher variability in the adsorption data obtained at high concentrations (the variation coefficient was near 20% in some cases), since the diffusion process through the pores in the surface is influenced by the surface roughness and the shape, quantity, and volume of the cavities and pores in the beads. The difficulty of obtaining adsorbent material with homogeneous roughness and pore shape is evident; this fact would help to explain our results. The Elovich kinetic model, which can study the adsorption rate based on adsorption capacity on heterogeneous surfaces, also offers a good adjustment to our data (Table 4).

Altogether, the obtained adsorbent structure after the acid treatment, with a higher surface area and pore volume as confirmed by BET analyses and observed by SEM, allowed the adsorption of a greater number of molecules of the azo dye under study and a more efficient removal than similar adsorbents with lower porosity, as reported in this and in previous research in our laboratory [9].

### 3.5. Adsorption Isotherms

In order to obtain the adsorption isotherms, equilibrium concentration and adsorption capacity data are necessary. The extraordinary elevated capacity to eliminate all the dissolved dye in the solutions made it necessary to widen the RB5 concentration range and time to fully characterize the adsorbent behavior (Figure S9), since nearly 100% of dye elimination was achieved in the conditions initially foreseen. Then, at 298.15 K, it was necessary to include an extra concentration, RB5 500 mg/L, and to extend the experiment procedure for 240 h (10 days) to achieve equilibrium. Higher RB5 concentrations were included at 318.16 K: 1000, 1500, and 2000 mg/L, for 10 days. The experimental isotherms can be found in Figure S9, which has been split in (a) and (b) so that the different concentration ranges can be observed adequately. 

**Figure 8:**
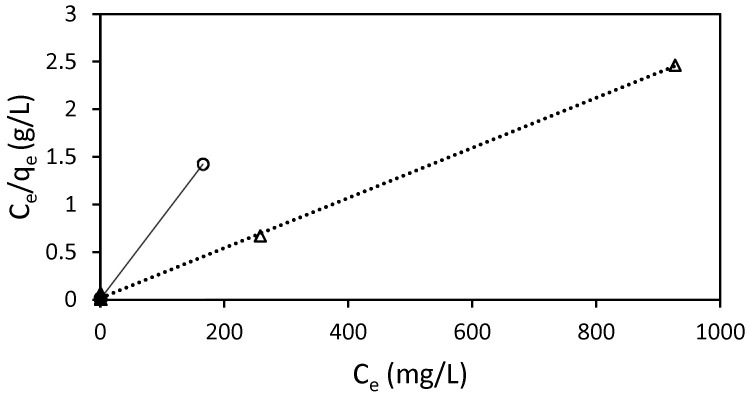
Langmuir isotherm for the adsorption of RB5 by 15 mg of ST-Ch-DES beads at 298 K (○) and 318 K (∆), and pH 6.

The adsorption of RB5 onto Ch-DES exhibited a favorable fit to the Langmuir isotherm model (R^2^ > 0.999) (Table 5, Figure 8), suggesting the arrangement of a monolayer of dye molecules on uniform distribution of sorption sites in the adsorbent, with no interactions among adsorbed molecules [25]. The other models considered showed much weaker correlations (R^2^ < 0.6713, Table 5) than the Langmuir one, so they were not considered to rationalize the adsorption process.

The separation factor constant, *R_L_*, allows us to estimate if the adsorption process is favorable or not, as it measures how suitable the adsorbent is. The obtained values at both temperatures were very low, 0 < *R_L_* < 1 (Table 5), so the adsorption process was favorable [46], in our conditions, according to the Langmuir adjusted data. The correlation between *K_L_* and the features of the adsorbent, such as surface area and pore volume, suggests that a larger surface area and higher pore volume lead to an increased adsorption capacity. Our results on ST-Ch-DES at 298 K show that this parameter was higher in this adsorbent than in the previous adsorbent Ch-DES, used in other dyes [9]. This is consistent with the increase in the pore volume and the BET surface area, as well as the micropore surface area, in the acid-treated adsorbents, as previously described in this work.

It should be mentioned that most of the obtained data were total adsorption values, instead of equilibrium values. The high adsorption capacity rendered it very difficult to obtain reliable equilibrium data, since the elevated contact time and the extremely high dye concentrations necessary to approach such equilibrium points were not realistic from an operational perspective.

### 3.6. Reusability of the Adsorbent

Given the high efficiency of the ST-Ch-DES as an adsorbent, it was of most interest to evaluate the potential for reuse of the material, allowing us to obtain a cost-effective process and recyclability of the materials involved, in view of adapting novel wastewater depuration processes by the textile industry. Firstly, we tried to separate the adsorbed dye ensuring the adsorbent integrity in the solvents. Previous solubility tests are summarized on Table S3.

#### 3.6.1. Desorption

**Figure 9:**
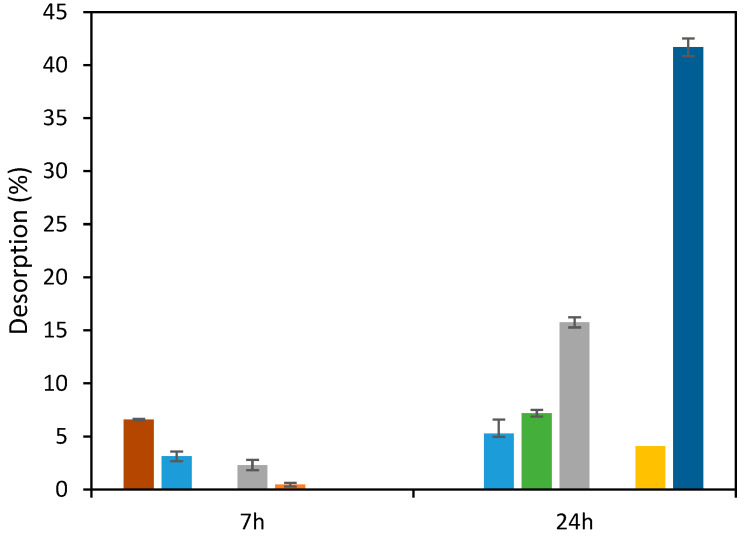
Results of desorption of RB5 from ST-Ch-DES beads applying alkaline or acid solvents at varying duration (7 h or 24 h), concentrations, and temperatures: NaOH 0.1M (■), NaOH 1M (■), NaOH 2.5 M (■), KOH 1 M (■), H_2_SO_4_ 0.1 M (■) (all at 298.16 K, 350 rpm); at T < 298.16 K (range: 288.16–295.16 K): NaOH 0.1 M (■) and NH_4_OH (■).

Alkaline solvents, namely, NaOH, have been used for desorption of anionic dyes as described by others [28,47]. Then, NaOH was tested to desorb RB5 from the ST-Ch-DES beads. However, the desorption achieved was only partial, increasing with time and alkaline solvent concentration (Figure 9). Interestingly, when the temperature was below 298.16 K, the desorption rate was higher, the value achieved in 7 h was 6.6%, twice the value at 298.16 K, 3.1%, and practically equals the desorption obtained after 24 h, 5.3%. Another alkaline solvent, NH_4_OH, was essayed, also at low temperature (range 288–295 K), with a better performance, since the desorption obtained was 41.7% (Figure 9). Desorption was negligible using sulfuric acid.

Hydroxyl ions and the anionic dye molecules compete among themselves, which might be responsible for the elution of RB5, as reported [28,47]. However, the adsorption of RB5 onto ST-Ch-DES beads seems stronger, possibly more difficult to desorb owing to the higher porosity and more cavities where the dye remains attached even after a long treatment with a strong alkali. 

The adsorbents treated with NaOH 0.1 M and H_2_SO_4_ 0.1 M for desorption were tested for reuse with a fresh RB5 solution. When NaOH 0.1 M was previously used, the removal of RB5 from the solution decreased from 99.7% to 26.4% (Figure S10). On the other hand, when H_2_SO_4_ 0.1 M was previously used, the removal of RB5 from the solution also decreased but still removed around 41%. These findings indicate that the adsorption capacity of RB5-adsorbed ST-Ch-DES beads experienced a subsequent enhancement following the acidic treatment. This improvement can be attributed to the protonation of the amine groups present in chitosan that enable stronger electrostatic interactions with the anionic dye [48]; hence, it could be used at half its initial capacity for one more time. A third use of this adsorbent only achieved around 13% RB5 removal.

#### 3.6.2. Regeneration

One of the most useful findings in this study is the capacity of the material to be regenerated and reused at a very high efficiency, complying with one of the main recommendations to ensure the adsorbent’s economic feasibility and industrial viability by improving the reusability of the beads [49].

**Figure 10:**
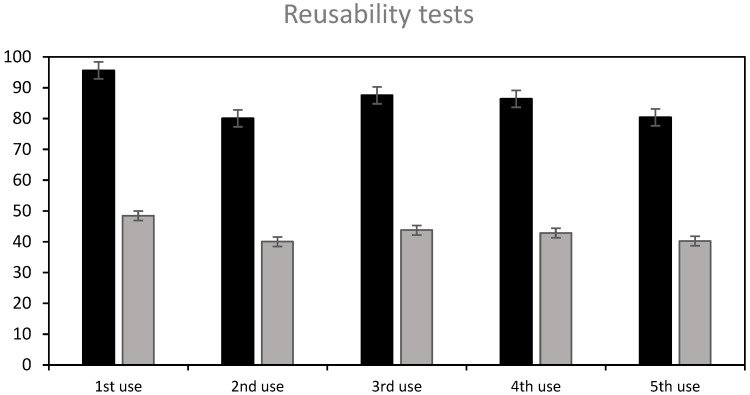
Reusability of partially NaOH-desorbed and acid-regenerated ST-Ch-DES beads, after four complete cycles of use-partial desorption–regeneration. The adsorption tests were carried out at 150 mg/L initial RB5 concentration, 15 mg of adsorbent, 350 rpm, and 7 hours contact time: (■) dye removal %, (■) adsorption (mg/g).

The following treatment sequence was applied: the beads were firstly used to adsorb RB5; then, they were treated with NaOH 0.1 M (7 h) to achieve a partial desorption, washed with water, regenerated with H_2_SO_4_ 0.1 M for 5 h so as to ensure that amphoteric groups are protonated, again washed with water, and finally reused to adsorb the dye. Figure 10 shows the results: the removal efficiency achieved in the subsequent uses was around 84% on average, after 7 h contact time, and the adsorption capacity, around 42 mg/g, was similar to the original ST-Ch-DES beads, 50.7 mg/g, at the fixed dye concentration level (150 mg/L). The beads were efficient during at least five uses, as shown in Figure 10, and stable after the four complete cycles of desorption–regeneration applied. Reuse of the beads was still possible at a lower efficiency (25% dye removal) without any further regeneration treatment (Figure S10). 

Therefore, regeneration of the ST-Ch-DES beads with H_2_SO_4_ treatment, applied after partial desorption with NaOH, was achieved, and this enabled further use of the adsorbent at an efficient removal rate. Therefore, these capacities may accomplish the industry requirements for a green cost-effective treatment of wastewater, in the context of a circular economy.

### 3.7. Possible Adsorption Mechanism

The ST-Ch-DES beads showed a notable improvement as an adsorbent for the azo dye RB5, as described throughout this study. The highest swelling behavior and the increased porosity and surface area, together with the protonation, enabled this extraordinary capacity, and the ionic linkages formed between the protonated amine groups (−NH_3_^+^) of chitosan and SO_4_^2−^ ions of sulfuric acid, as shown from our previously described FTIR results, probably increased the chemical stability of the beads in acidic medium, as also reported by others [24]. As a result, a greater number of adsorption sites were available to support the RB5 adsorption from aqueous solutions. 

The FTIR spectra of ST-Ch-DES and ST-un-Ch after RB5 adsorption showed the typical bands of both adsorbents together with the bands characteristic of the colorant RB5 (Figure S11). Slight differences were observed after adsorption between 2848 and 2963 cm^−1^, stretching vibration of C-H groups, and also around 3300 cm^−1^, stretching vibration of O-H and N-H groups, indicating that the dye RB5 was assimilated on the Ch-DES beads. There are no evident new peaks, and the ones in the 1570–400 cm^−1^ region decreased after adsorption, especially in the ST-Ch-DES beads, which would mean that the adsorbent-dye interaction is essentially physical [26].

The adsorption follows the pseudo-second-order kinetic model at the lowest RB5 concentrations (25, 50, and 100 mg/L), where the surface adsorption controlled by chemical adsorption, probably dependent on the electrostatic forces, namely, the interaction between the protonated amine groups (–NH_3_^+^) of chitosan and the sulfonate groups in the anionic diazo dye RB5, was the main adsorption mechanism. At higher RB5 concentrations (150–250 mg/L), the intraparticle diffusion model better explains the process: fast diffusion on the surface during a short time, followed by a gradual adsorption step with slow particle diffusion between the RB5 films formed during the first stage and the interaction with the porous structure in the beads core, and final stabilization in the adsorption rate when practically total adsorption of the dye molecules in the solution has occurred. 

### 3.8. Comparison of Ch-DES Adsorption Capacities

Differences among adsorbent materials with variable capacity values (*q_m_*) are reported in the literature. The *q_m_* (Langmuir) at 298.16 K and pH 6 obtained for RB5 in this work was 116.78 mg/g, clearly superior to other adsorbents: fungal biomass (26.95 mg/g) [50], commercial granular activated carbon (12.25 mg/g) [51], biochar from gasification of wood waste (35.67 mg/g) [52], Pb-doped ZnO nanoparticles by sol-gel technique (29.06 mg/g) [53], mushroom waste (14.62 mg/g) [54], textile scraps from the clothing industry treated by pyrolysis and chemical activation with K_2_CO_3_ (10.3 mg/g) [55], and banana peel powder (49.2 mg/g at pH 3) [56]. Our adsorbent was even more efficient in removing RB5 than a commercial activated carbon (94.7 mg/g) [57].

Other adsorbents were reported to reach similar capacities, such as macadamia nutshell chemically activated and converted to magnetic mesoporous carbon (123.51 mg/g) [51], and higher adsorption capacities, when comparing our value at 298.16 K: commercial powdered activated carbon (193 mg/g, pH 4) [58], chitosan/polyamide nanofibers (205.2 mg/g, 298 K), cellulose crosslinked with polyethyleneimine and magnetic nanoparticles (330 mg/g) [59], and polyethylene terephtalate (PET) waste bottles as substrate for titanium dioxide films (155.04 mg/g) [60]. At higher temperatures, our adsorbent even surpassed these efficiencies towards RB5, with *q_m_* values of 379.9 mg/g at 318.16 K.

It should also be mentioned that there are methods different from adsorption that try to remove Reactive Black 5 from aqueous matrices, albeit with an efficiency lower than the one achieved with these adsorbents. For example, there are photochemical approaches that degrade the dye, achieving a 90.7% removal [61], or biochemical methodologies that eliminate 67.4% of a mixture of dyes that contains Reactive Black 5 [62].

## 4. Conclusions

In conclusion, this study presents a novel approach for the preparation of chitosan-based beads aimed at enhancing their effectiveness and reusability in textile dye adsorption. The treatment of chitosan beads with sulfuric acid and deep eutectic solvent (DES) demonstrated a remarkable improvement in their adsorption capacity towards the diazo dye Reactive Black 5. The acid-treated chitosan-DES beads exhibited enhanced porosity, surface area, and swelling behavior while maintaining stability across a wide range of pH conditions and temperatures. Our findings reveal that the modified chitosan beads effectively removed nearly all of the colorant from aqueous solutions within the investigated concentration range, achieving full decolorization up to 250 mg/L. Remarkably, the material demonstrated excellent reusability, allowing for multiple cycles of regeneration and dye recovery while maintaining optimal efficiency. Furthermore, the adsorption process followed the Langmuir isotherm model, indicating a favorable adsorption mechanism. Quantitative data analysis further showed that the adsorption efficiency was exceptionally high under acidic conditions and remained significant up to pH 8, which aligns with the pH range typically observed in textile industry dyeing discharge. The pseudo-second-order kinetic model and the intraparticle diffusion model provided valuable insights into the adsorption process. Additionally, we observed that increasing the temperature resulted in a moderate increase in adsorption efficiency, significantly accelerating the process by up to 10 times at elevated dye concentrations. The remarkable performance and reusability of the chitosan-DES beads makes them an environmentally sustainable material with vast potential for various green technology applications, particularly in the improvement of textile industrial water remediation.

## Figures and Tables

**Table 1:** Outline of the conditions for the experimental tests.

Series	Dye Concentration (mg/L)	Adsorbent	Adsorbent Weight (g)	Temperature (K)	pH
**0**	150	un-Ch Ch-DES ST-un-Ch ST-Ch-DES	0.015	298.16	6.0
**1**	150	ST-Ch-DES	0.005, 0.010, 0.015, 0.025, 0.035	298.16	6.0
**2**	25, 50, 100, 150, 200, 250	ST-Ch-DES	0.015	298.16	6.0
**3**	25, 50, 100, 150, 200, 250	ST-Ch-DES	0.015	298.16, 318.16	6.0
**4**	150	ST-Ch-DES	0.015	298.16	3.6, 5.2, 6.0, 8.2, 9.2

**Table 2:** BET and BJH analysis related to the surface area and porosity of un-Ch, Ch-DES, ST-un-Ch, and ST-Ch-DES.

	un-Ch	Ch-DES	ST-un-Ch	ST-Ch-DES
BET surface area (m^2^/g)	0.34	0.15	2.27	1.97
pore volume (cm^3^/g)	3.6 × 10^−4^	1.5 × 10^−4^	20.4 × 10^−4^	18.4 × 10^−4^
pore size (nm)	4.16	4.05	3.60	3.73
micropore surface area (m^2^/g)	0.54	0.23	3.14	2.82

**Table 3:** Swellings of the unmodified chitosan beads and the DES-modified chitosan beads treated by the different protocols: soaked in water (W), soaked in sulfuric acid (A), and soaked first in water and then in sulfuric acid (W-A), monitored by weight (g) and diameter (mm) for each treatment.

Ch-DES		
Treatment	Swelling Ratio (%)	Diameter Increase (%)
W (H_2_O)	109.3 ± 2.1	22.4 ± 0.6
A (H_2_SO_4_)	149.4 ± 1.5	29.3 ± 0.9
W-A (H_2_O + H_2_SO_4_)	226.5 ± 2.5	44.2 ± 0.4
**un-Ch**		
W (H_2_O)	80.8 ± 1.2	26.8 ± 2.2
A (H_2_SO_4_)	128.5 ± 2.6	29.2 ± 1.5
W-A (H_2_O + H_2_SO_4_)	118.7 ± 2.0	29.9 ± 0.9

**Table 4:** Parameters of the kinetic models for RB5 adsorption on ST-Ch-DES beads.

Dye	RB5					
Concentration (mg/L)	25	50	100	150	200	250
*q_e_*, exp (mg/g)	8.57	16.92	34.73	50.76	69.45	83.35
Pseudo-first-order kinetic model						
*k*_1_ (1/min)	41.9 × 10^−3^	37.9 × 10^−3^	13.6 × 10^−3^	6.2 × 10^−3^	2.3 × 10^−3^	0.8 × 10^−3^
*q_e_* (mg/g)	9.56	17.15	32.71	48.51	56.77	64.50
R^2^	0.9968	0.9873	0.9957	0.9158	0.9960	0.9502
Pseudo-second-order kinetic model						
*k*_2_ (mg/(g min))	4.7 × 10^−3^	2.7 × 10^−3^	0.4 × 10^−3^	0.2 × 10^−3^	0.7 × 10^−4^	0.4 × 10^−4^
*q_e_* (mg/g)	9.91	19.13	42.07	58.65	78.03	86.34
R^2^	0.9938	0.9933	0.9972	0.9549	0.9738	0.9587
Elovich kinetic model						
*β* (g/mg)	0.4626	0.2488	0.109	0.0877	0.0718	0.0789
*α* (mg/(g min))	0.940	2.131	1.484	1.425	1.261	1.054
R^2^	0.9165	0.9198	0.9888	0.9424	0.9330	0.8938
Intraparticle diffusion kinetic model						
*k*_3_ (mg/(g min^1/2^))	0.526	0.9889	1.957	2.1609	1.9665	1.4123
*I*	2.624	5.585	3.931	3.364	5.692	9.935
R^2^	0.7955	0.8159	0.9403	0.9887	0.9830	0.9867

**Table 5:** Isotherm parameters for RB5 adsorption on ST-Ch-DES beads.

Bead Type: ST-Ch-DES			
Model	Parameters	298.16 K	318.16 K
Langmuir isotherm	*k_L_* (L/mg)	1.61	0.17
	*q_m_* (mg/g)	116.78	379.90
	*R_L_*	0.0024	0.0055
	R^2^	0.9999	0.9991
Freundlich isotherm	n	5.13	3.37
	k_F_	46.48	63.89
	R^2^	0.2988	0.4149
Temkin isotherm	B (J/mol)	10.89	44.00
	k_T_ (L/mol)	303.16	14.70
	R^2^	0.5453	0.6713
Elovic isotherm	k_e_	10.084	0.845
	q_m_ (mg/g)	36.589	134.572
	R^2^	0.2205	0.2574
Dubinin–Radushkevich isotherm	β ×10^−8^ (kJ/mol)	5.888	16.659
	E (kJ/mol)	0.291	0.173
	q_m_ (mg/g)	123.05	226.21
	R^2^	0.4087	0.3317

## Data Availability

The data presented in this study are available in the manuscript and Supplementary Materials.

## Supplementary Materials

The following supporting information can be downloaded at: https://www.mdpi.com/article/10.3390/molecules29071610/s1.

## Abbreviations

Unmodified chitosan beads (un-Ch), deep eutectic solvent (DES), DES-modified chitosan beads (Ch-DES), sulfuric-acid-treated unmodified chitosan beads (ST-un-Ch), sulfuric-acid-treated DES modified chitosan beads (ST-Ch-DES), Reactive Black 5 (RB5), Scanning electron microscopy (SEM), Barrett–Joyner–Halenda (BJH) pore size and volume analysis, Brunauer–Emmett–Teller (BET) surface area analysis, Attenuated Total Reflection (ATR) Spectroscopy, Fourier-transform infrared (FTIR) spectrum, choline chloride (ChCl).
