# Supplementary material for: Accessible Eco-Friendly Method for Wastewater Removal of the Azo Dye Reactive Black 5 by Reusable Protonated Chitosan-Deep Eutectic Solvent Beads
Source: Molecules. 2024 Apr 3;29(7):1610. doi: 10.3390/molecules29071610 (PMC11013712; doi:10.3390/molecules29071610)
Supplement: Supplementary file 1 [file molecules-29-01610-s001.zip › molecules-2925732-supplementary.docx]

Accessible eco-friendly method for wastewater removal of the azo dye Reactive Black 5 by reusable protonated chitosan- deep eutectic solvent beads

**Supplementary information**

(a)


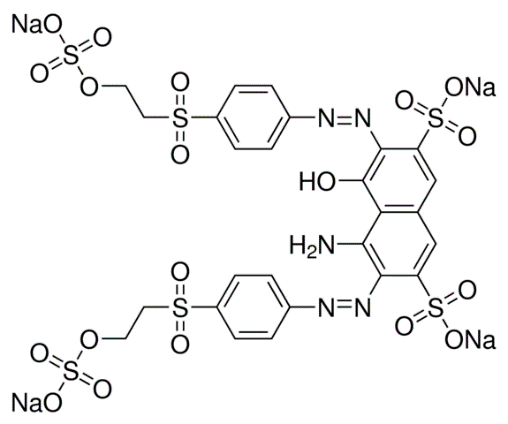

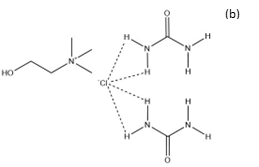

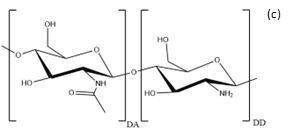


Figure S1: Structures of the dye RB5 (a), the proposed structure for choline chloride:urea 1:2 eutectic mixture (b), and chitosan structure (c).

Table S1: Compounds used in this study and specifications -(chemical name, supplier, purity provided by supplier and CAS number).

| **Chemical name** | **Supplier** | **Purity** | **CAS number** |
| --- | --- | --- | --- |
| Chitosan | Acros Organics | - | 9012-76-4 |
| Choline chloride | Acros Organics | 99% | 67-48-1 |
| Urea | Labkem | 98-102% | 57-13-6 |
| Reactive Black 5 | Sigma Aldrich | 50% | 17095-24-8 |
| NaOH | Fisher Scientific | ≥ 97% | 1310-73-2 |
| Ethanol absolute | Scharlau | 99.9% | 64-17-5 |
| Acetic acid glacial | Labkem | 99.8% | 64-19-7 |
| Sulfuric acid | Labkem | > 95% | 7664-93-9 |
| Hydrochloric acid | VWR | 37% | 7647-01-0 |
| Sodium nitrate | Labkem | >99.5% | 7631-99-4 |
| Ammonium hydroxide | Sigma-Aldrich | 25% NH_3_ basis | 1336-21-6 |
| KOH | Merck |  | 1310-58-3 |

*Calculation of deacetylation degree (DD) of chitosan*

The deacetylation degree (*DD*) was determined by Fourier Transform Infrared spectroscopy (FTIR), The equation (S1) was used for the determination of *DD* [1]:


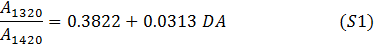


Where *DA* = acetylation degree (%); *DD*=100-*DA*; *A_1320_* = Area under the curve in the IR spectra band 1320 cm^-1^ ; *A_1420_* = Area under the curve in the IR spectra band 1420 cm^-1^ . The Gaussian model was used to obtain the information from the original spectra (areas centred on 1324.1 and 1421.7 cm^-1^).

*Adsorption experiments*

The data were presented as mean values ± standard error of the mean calculated as follows (eq S2):


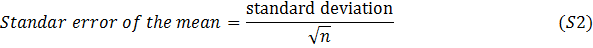


Where *n* is the sample size (number of individual observations) and standard deviation is calculated as follows (eq S3):


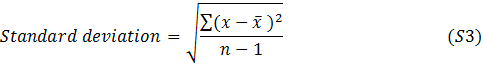


Where is the mean value of the *n* individual observations (*x*).


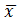

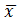


*Adsorption kinetics models:*

The adsorption kinetic models used were pseudo-first and pseudo-second order models (Eq. S4 and S5, respectively), Elovich kinetic model (Eq. S6) and intraparticle diffusion (Eq. S7)


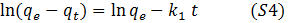

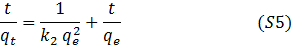

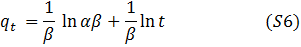

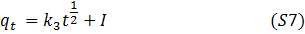


Where: *q_e_* and *q_t_* are the adsorbed dye at equilibrium and time *t*; *K_1_* (min^-1^) denotes the pseudo-first-order rate constant, *K_2_* (g mg^-1^min^-1^) denotes the pseudo-second-order rate constant; *K_3_* symbolizes the intraparticle propagation rate constant; *I* is a constant and is associated with the boundary layer thickness [2]; *α* implies the adsorption rate at time = 0 min; *β* represents the coverage extent.

2.6. Adsorption isotherm

Langmuir, Freundlich, Temkin, Elovich and Dubinin–Radushkevich isotherm models have been used to study the affinity, trend, and adsorption mechanism of the dye towards sorbent. Equations S8 – S15 represent the mentioned models, respectively:


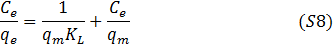

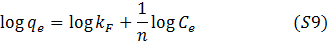

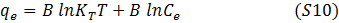


𝐶_𝑒_ is concentration of adsorbate at equilibrium (final concentration of solute (mg/L)/liquid-phase concentrations of dye at equilibrium); *q_e_* is the amount of dye adsorbed at equilibrium (mg/g); 𝐾_𝐿_ (L/mg or L/mol) is Langmuir constant related to adsorption capacity (mg g^−1^), which can be correlated with the variation of the suitable area and porosity of the adsorbent, implying that large surface area and pore volume will result in higher adsorption capacity. It is calculated from the intercept of the different straight lines (linear plot of *C_e_*/*q_e_* vs. *C_e_*) at different temperatures. 𝐾_𝐹,_ Freundlich constant (L/g), is related to the adsorption capacity, and 1/𝑛 is a function of the strength of adsorption, indicating the affinity between the adsorbent and adsorbate. A value of 1/*n* below unity would imply a chemical process, and above unity, a physical process; it also indicates the heterogeneity of the surface: the more heterogeneous the surface, the more the 1/*n* value approaches zero. 𝐾_𝑇_ is Temkin isotherm constant (L g^−1^). *T* (K) is the absolute temperature. *R* represents the universal gas constant (8.314 J mol^-1^K^-1^). *B = RT/b* is a constant which is related to the heat of adsorption (J mol^−1^); *q_m_* is the maximum amount of dye adsorbed (mg/g) / maximum monolayer adsorption capacity (calculated from the slope of the different straight lines at different temperatures).

The value of the dimensionless constant called the separation factor 𝑅_𝐿_ , based on the following equation (S11) could be used to predict the degree of favourability of the Langmuir isotherm for equilibrium data:


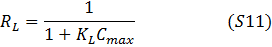


𝐾_𝐿_ is Langmuir constant and 𝐶*_max_* is the highest initial dye concentration. 𝑅_𝐿_ values indicate the adsorption to be unfavourable when 𝑅_𝐿_ > 1, linear when 𝑅_𝐿_ = 1, favourable when 0 < 𝑅_𝐿_ < 1, and irreversible when 𝑅_𝐿_ = 0 [19].

Dubinin-Radushkevich isotherm is expressed as follows (Eq. S12) :


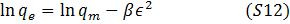

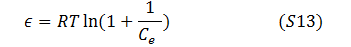

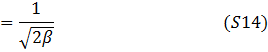


𝜖 is Polanyi potential, 𝛽 is Dubinin-Radushkevich constant, 𝑅 is gas constant (8.31 Jmol^−1^ K^−1^), 𝑇 is absolute temperature, and 𝐸 is mean adsorption energy.

The Elovich isotherm linear form is expressed as follows (Eq. S15):


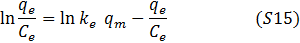


Elovich maximum adsorption capacity and Elovich constant can be calculated from the slope and intercept of the plot of ln(*q_e_*/*C_e_*) vs *q_e_*.


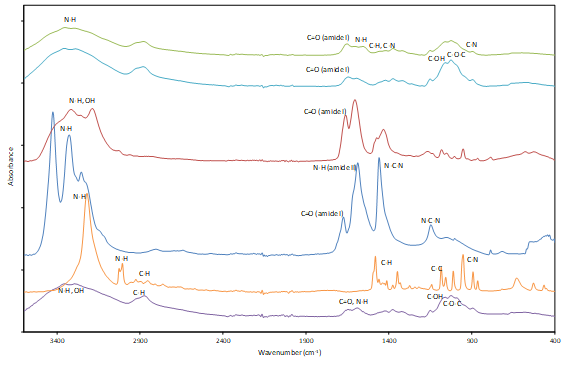


Figure S2: Fourier transform infrared (FTIR) spectra of the starting materials chitosan (**─**), choline chloride (**─** ChCl), urea (**─**), Deep eutectic solvent choline chloride:urea (**─** DES ChCl:U) 1:2,and the chitosan-based adsorbents unmodified chitosan (**─** un-Ch) beads, DES-modified chitosan (**─** Ch-DES) beads.

Table S2: Main functional groups observed in the individual components of un-Ch and Ch-DES beads by FTIR analysis.

| Chitosan | | |
| --- | --- | --- |
| Region (cm^-1^) | Functional groups | Comments |
| 3294-3352 | N-H and O-H | N-H and O-H stretching vibration |
| 2875-2920 | C-H | CH, CH_2_ and CH_3_ |
| 1025, 1059 | C-O-C and C-OH | Stretching vibrations |
| 1592, 1645 | N-H, C=O | N-H bending, C=O stretching vibration Amide I (CONH_2_) and II (N-H). Ionic cross-linking (acetic acid) |
| Choline chloride | | |
| Region (cm^-1^) | Functional groups | Comments |
| 3006-3026 3219 | N-H | Stretching vibration |
| 1413-1481 | C-H | Bending of aliphatic C-H groups |
| 1480-1390 | N-H | N-H vibrations in quaternary ammonium groups |
| 1200-880 | C-N | Asymmetric C-N stretching vibration |
| 2854-2951 | C-H | Stretching vibrations of C-H in CH_2_ and CH_3_ |
| Urea | | |
| Region (cm^-1^) | Functional groups | Comments |
| 3425-3326 | N-H | Asymmetric N-H stretching vibration of NH_2_ groups |
| 3228-3254 | N-H | Symmetric N-H stretching vibration of NH_2_ groups |
| 1674 | C=O | Stretching vibration of C=O in amide group (CONH_2_) (amide I band) |
| 1587 | N-H | Bending of N-H bond in NH_2_ groups (amide II band) |
| 1459, 1147 | N-C-N | Asymmetric and symmetric stretching vibration of N-C-N bonds |
| DES ChCl:Urea (1:2) | | |
| Region (cm^-1^) | Functional groups | Comments |
| 3500-3000 |  | Broadening of the bands due to hydrogen bonds between acceptor and donor |
| 3388 and 3314 | N-H and O-H | Stretching vibration of N-H and O-H bonds |
| 3256 and 3187 | N-H | Stretching vibration of N-H |
| 1660 | C=O | Shift to lower frequencies (C=O stretching vibration band of amide group CONH) |

**Un-Ch**

**Un-Ch**


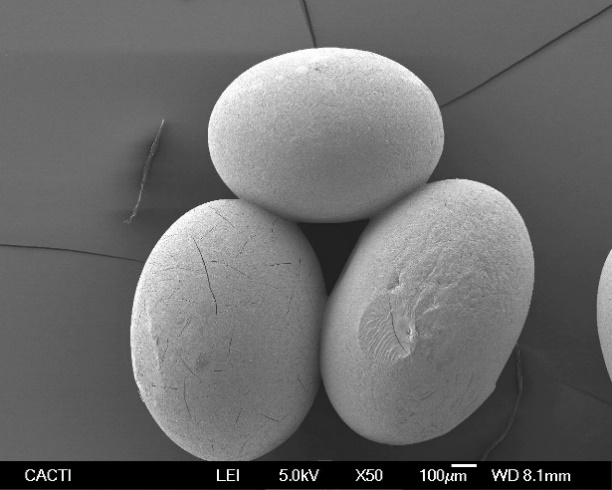

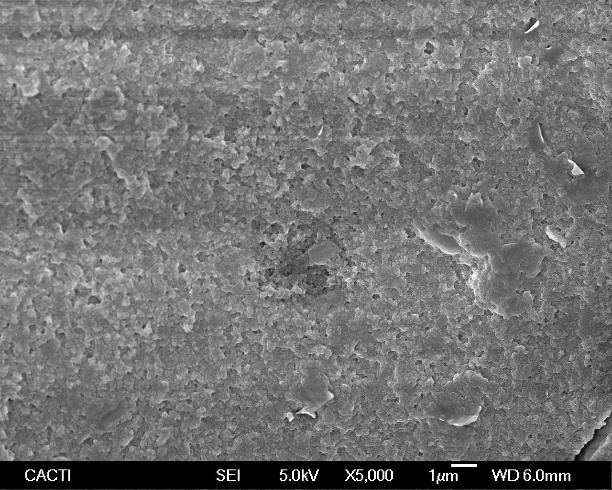


**ST-Un-Ch**


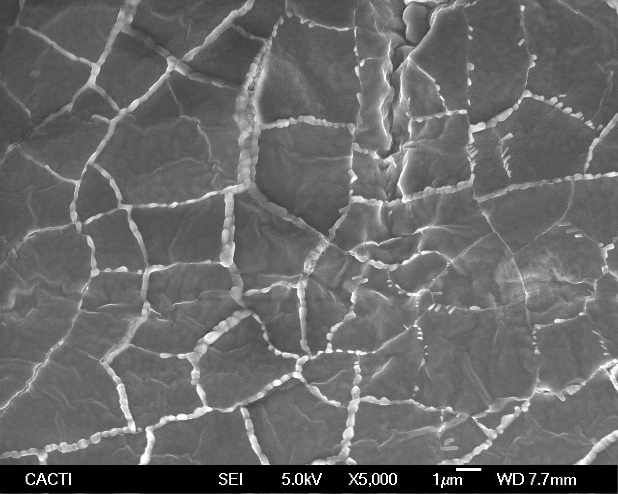


**ST-Un-Ch**


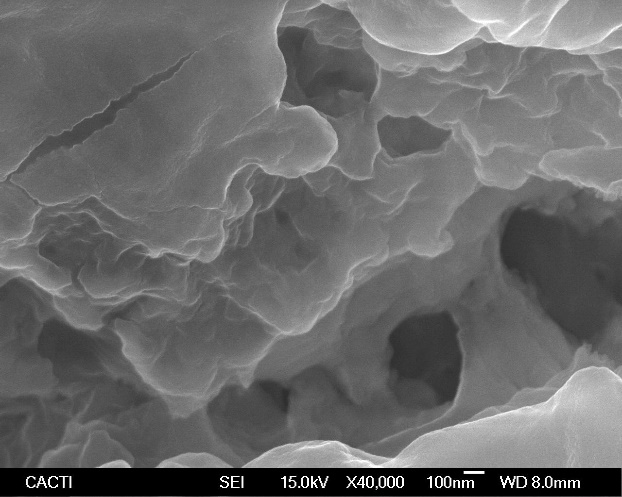


**ST-Ch-DES**


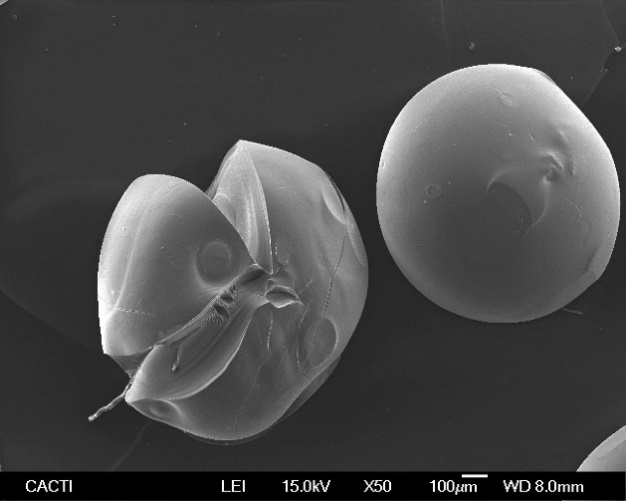


**ST-Ch-DES**


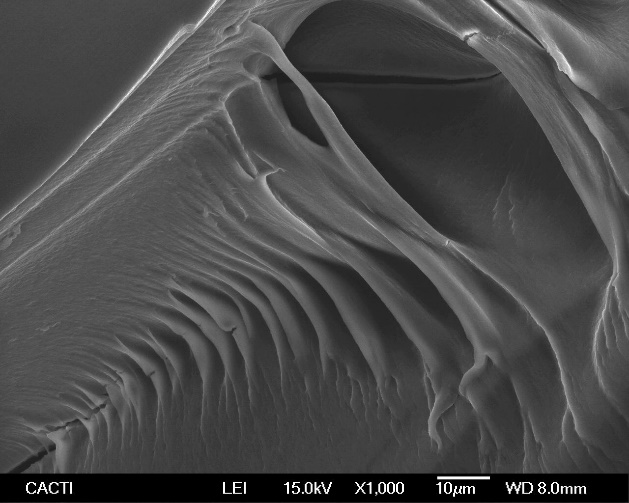


**ST-Ch-DES**


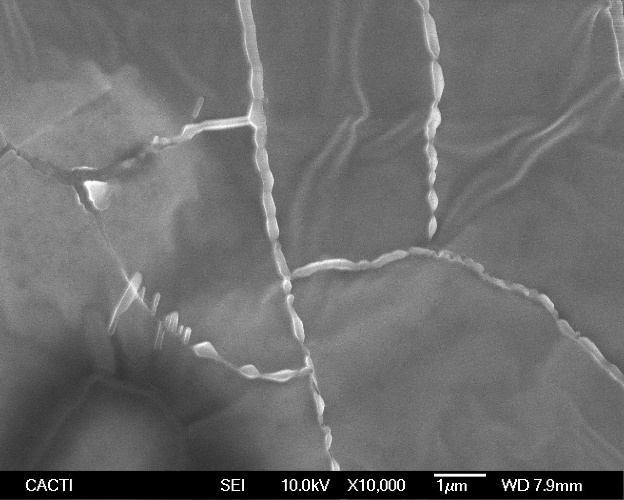


**ST-Ch-DES**


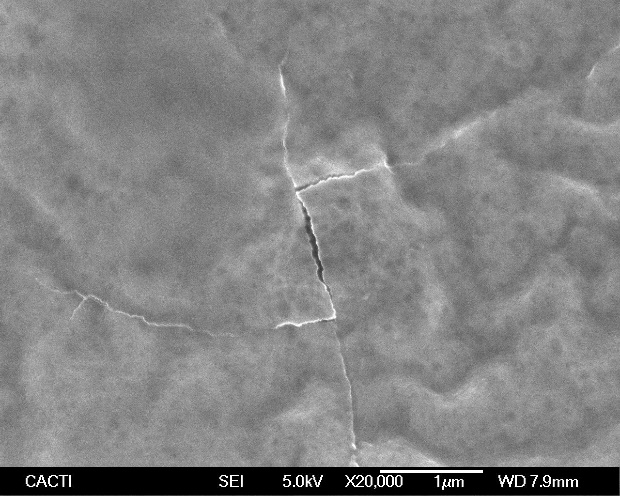


**ST-Ch-DES**


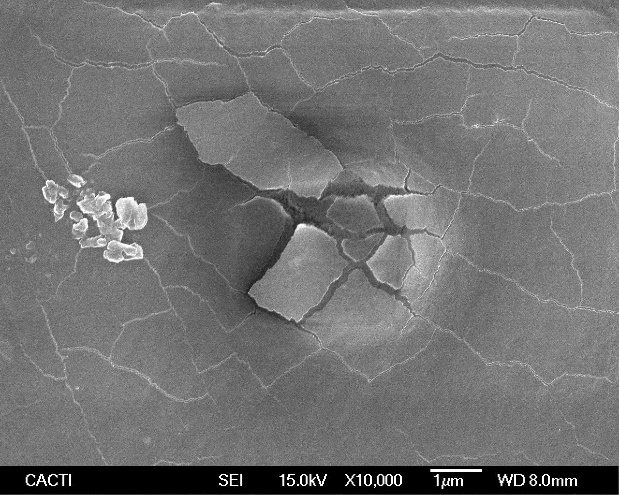


**ST-Ch-DES**


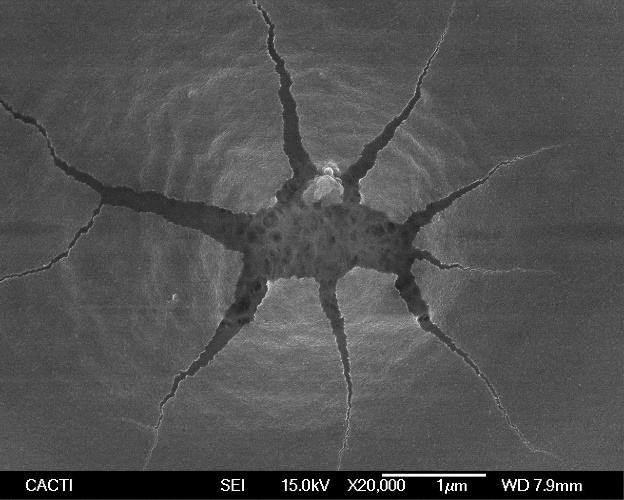


Figure S3: Scanning electron microscopy (SEM) images of the adsorbents unmodified chitosan (un-Ch), chitosan-DES (Ch-DES), sulfuric acid-treated unmodified chitosan (ST-un-Ch) and sulfuric acid-treated chitosan-DES (ST-Ch-DES) beads.


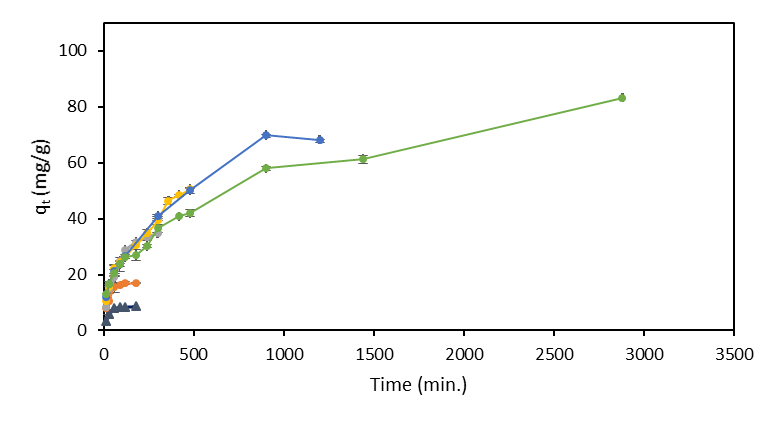


Figure S4: Effect of Reactive Black 5 concentrations and contact time on the amount of dye adsorbed (q_t_) at 298 K and pH 7 by 15 mg of Ch-DES beads: (▲) 25 mg/L, (●) 50 mg/L, (●) 100 mg/L, (●) 150 mg/L, (●) 200 mg/L, (●) 250 mg/L.


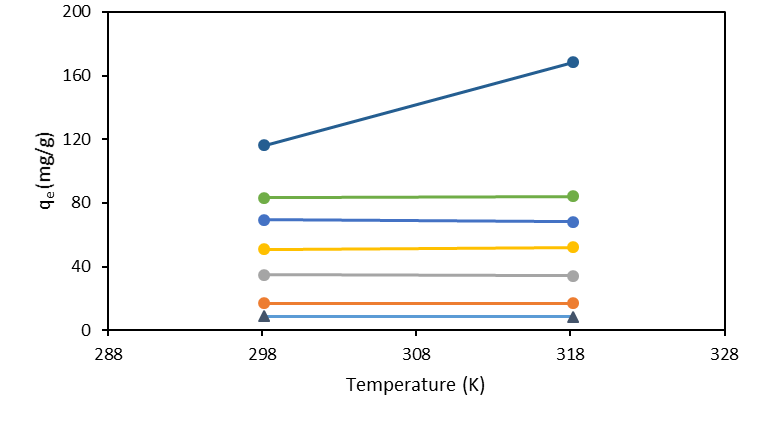


Figure S5: Effect of the different temperatures on the adsorption capacity of the dye RB5 by 15 mg of ST-Ch-DES on 5 mL of RB5 solutions at initial concentrations of 25-250 mg/L: (▲) 25 mg/L, (●) 50 mg/L, (●) 100 mg/L, (●) 150 mg/L, (●) 200 mg/L, (●) 250 mg/L, (●) 500 mg/L.


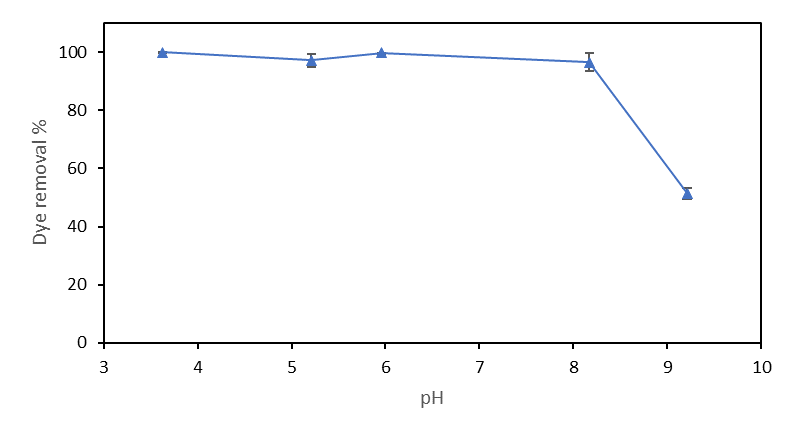


Figure S6: Effect of the different pH values on the removal efficiency of the dye RB50 by 15 mg of ST-Ch-DES on 5 mL of RB5 solutions at initial concentrations of 150 mg/L, after 480 minutes, at 298 K (error bars represent SEM).


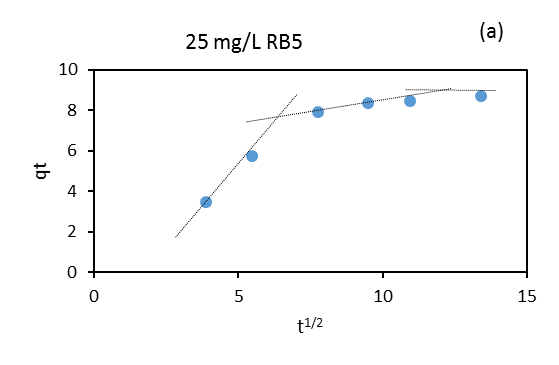

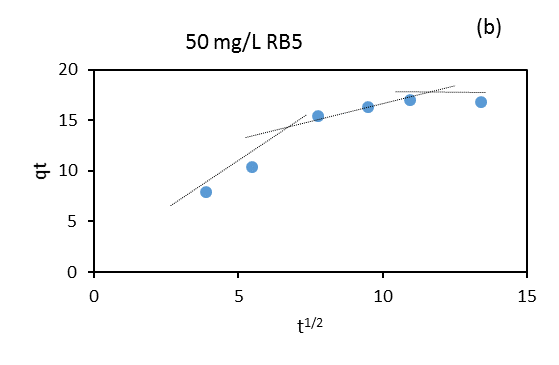

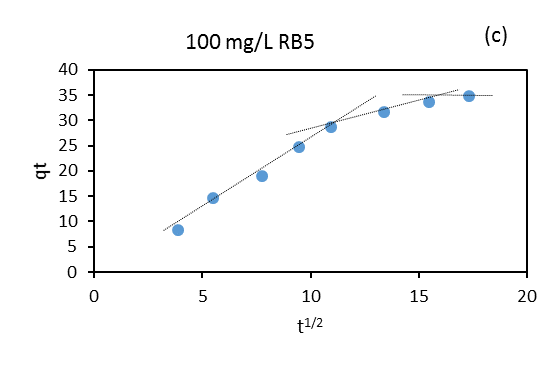


Figure S7: intraparticle diffusion model (Weber-Morris model) for adsorption of RB5 at initial concentration of 25 mg/L (a), 50 mg/L (b), 100 mg/L (c), at 298 K by 15 mg of Ch-DES beads.


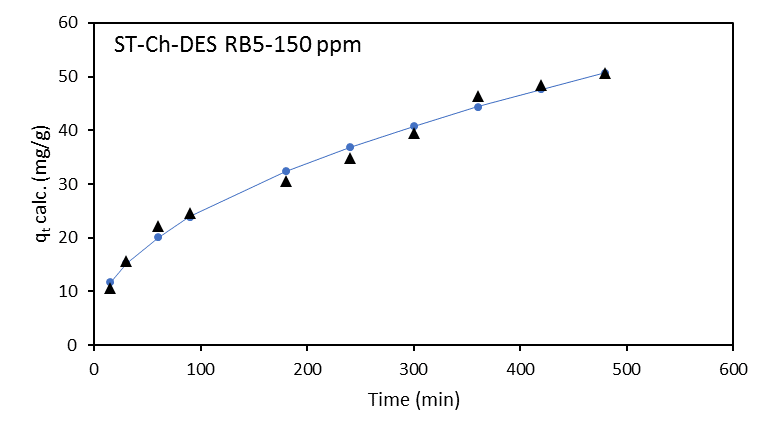

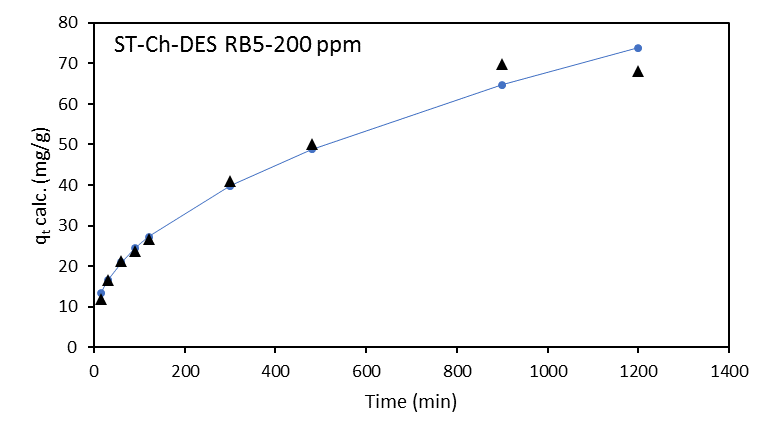

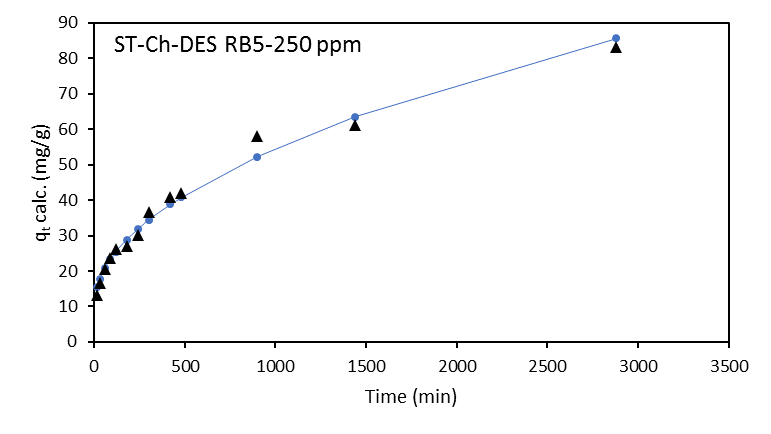


Figure S8: Adsorption kinetics plots for RB5 adsorption obtained at 298 K by 15 mg of ST-Ch-DES beads at different initial RB5 concentrations (150, 200 and 250 mg/L): experimental values obtained (▲), and calculated values for the intraparticle diffusion (Weber-Morris) model (●).

| 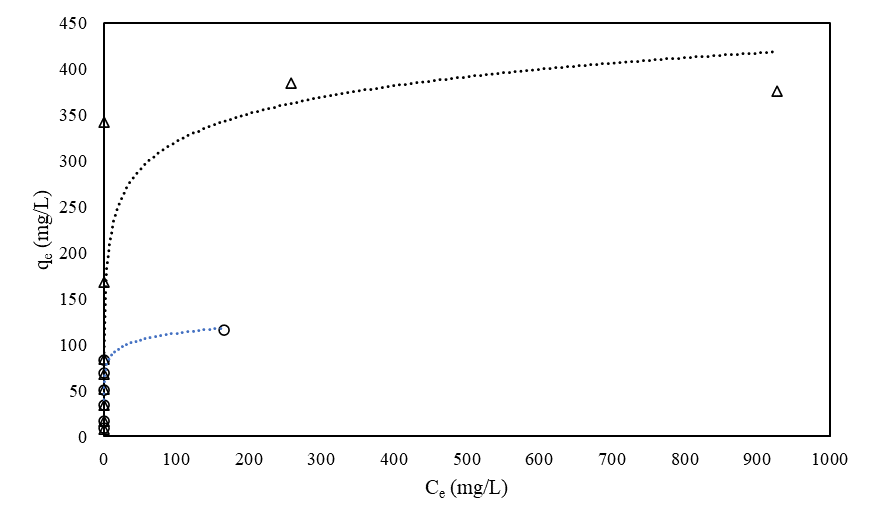 |
| --- |
| a) |
| 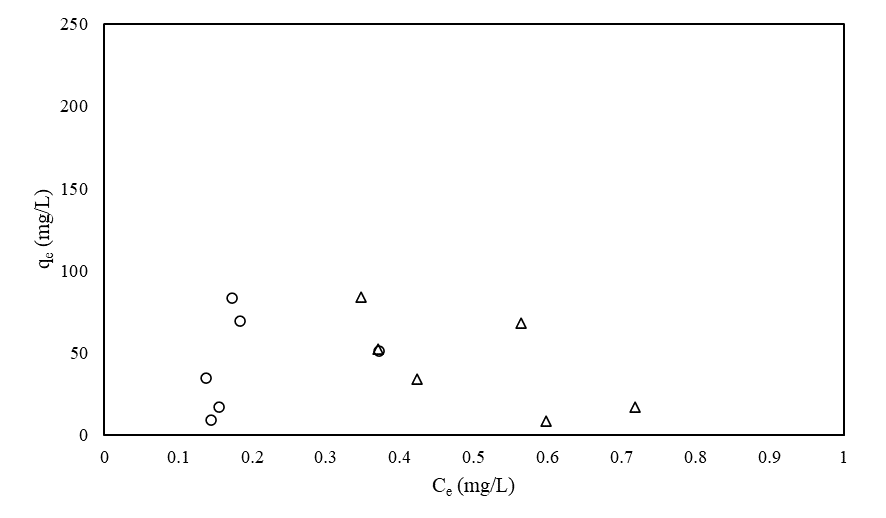 |
| b) |

Figure S9: Non-linear isotherms of RB5 adsorption on ST-Ch-DES beads at different temperatures: (○) 298.15 and (∆) 318.15 K. Figure a) shows the entire range of concentrations and b) depicts the low concentration regime.

Table S3. Experimental Ce and qe values at the two considered temperatures.

| 298.15 K | | 318.15 K | |
| --- | --- | --- | --- |
| Ce | qe | Ce | qe |
| 0.14476747 | 8.67561441 | 0.5970131 | 8.56555732 |
| 0.15470693 | 16.8115665 | 0.71794325 | 16.9146666 |
| 0.13814116 | 34.7338742 | 0.42307247 | 34.1330385 |
| 0.37171857 | 50.7578662 | 0.37006199 | 51.9816144 |
| 0.18405202 | 69.4453683 | 0.56388155 | 68.1129736 |
| 0.17237709 | 83.3469684 | 0.34686991 | 84.0897616 |
| 165.632456 | 116.341142 | 0.80298089 | 168.483921 |
|  |  | 1.21657304 | 342.354018 |
|  |  | 258.152303 | 384.855602 |
|  |  | 927.411896 | 376.525854 |

*Solubility tests*

Table S4 shows the solubility of ST-Ch-DES in the different solutions tested, and the potential RB5 desorption. They were insoluble in aqueous solutions of ethanol, pure ethanol, acetone, all the alkaline solutions and also in acid solutions, but were partially dissolved and disagregated in 0.1 M HCl. Alkaline solutions showed the highest potential for RB5 desorption from the ST-Ch-DES beads.

Table S4: Solubility and RB5 desorption behaviour of ST-Ch-DES (RB5 previously adsorbed) in different solutions (0.015 g of the beads into 5 mL of each solution, stirred for 24 hours).

| Solution | Solubility | Potential RB5 desorption |
| --- | --- | --- |
| Acetic acid 2% | insoluble | transparent |
| 0.1M H_2_SO_4_ | insoluble | slightly coloured |
| 0.1M HCl | soluble | coloured-beads disgregation |
| 0.01M HCl | insoluble | slightly coloured |
| 0.1M NaOH | insoluble | coloured |
| 1M NaOH | insoluble | coloured |
| 2.5M NaOH | insoluble | coloured |
| 1M KOH | insoluble | coloured |
| NH_4_OH | insoluble | coloured |
| Ethanol | insoluble | transparent |
| Ethanol:H_2_O (8:2) | insoluble | transparent |
| Acetone | insoluble | transparent |

*Reusability tests*

The used adsorbent was tested for reuse with fresh RB5 solutions. After the first use, the beads were rinsed with water, without further treatment or desorption. During the second use, the adsorbent scarcely maintains the adsorption capacity, only eliminating 25% of the dye RB5 from the aqueous solution.

The results obtained after reuse of untreated (not desorbed) ST-Ch-DES beads are shown on Fig. S10.


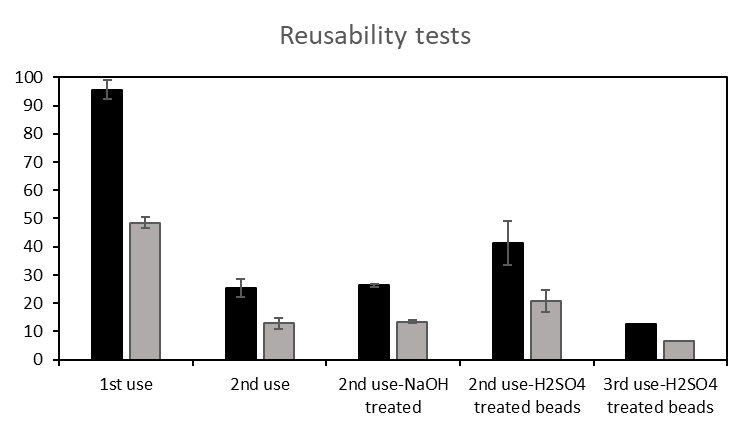


Figure S10: Results of reusability of not desorbed and partially-desorbed (NaOH treated beads and H_2_SO_4_ treated beads) ST-Ch-DES beads, at 150 mg/L initial RB5 concentration, 15 mg of adsorbent, 298 K, 350 rpm, and 7 hours contact time: (■) dye removal %, (■) adsorption (mg/g).

Figure S11: Fourier transform infrared (FTIR) spectra obtained after the adsorption of the dye RB5 onto acid treated unmodified chitosan (**─** ST-un-Ch-RB5) beads and acid treated modified chitosan (**─** ST-Ch-DES-RB5) beads compared to the spectra of both adsorbents before adsorption (**─** ST-un-Ch), (**─** ST-Ch-DES), and the spectra of the dye RB5 (**─**).


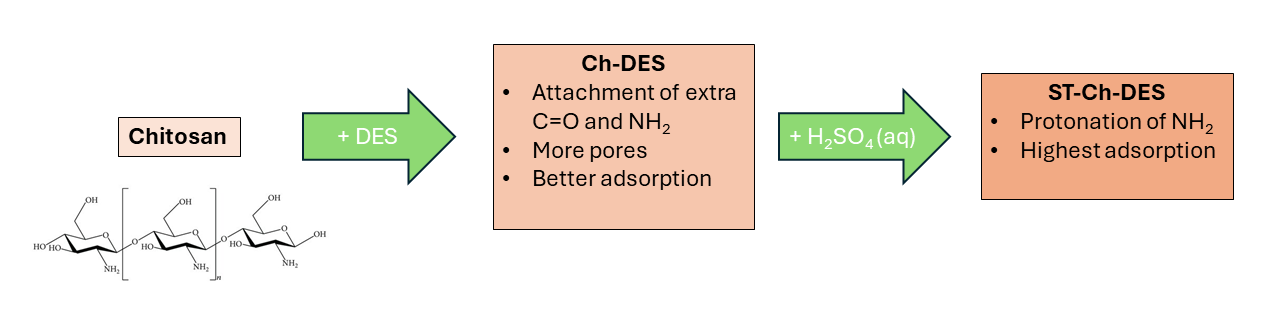


Figure S12. Schematic representation of the modifications performed on the beads.

**References**

1. Brugnerotto, J., Lizardi, J., Goycoolea, F. M., Argüelles-Monal, W., Desbrieres, J., & Rinaudo, M. An infrared investigation in relation with chitin and chitosan characterization. *Polymer*, 2001 *42*(8), 3569-3580. https://doi.org/[10.1016/S0032-3861(00)00713-8](http://dx.doi.org/10.1016/S0032-3861(00)00713-8)
2. Soliman, N.K.; Moustafa, A.F.; El-Mageed, H.R.A.; Abdel-Gawad, O.F.; Elkady, E.T.; Ahmed, S.A.; Mohamed, H.S. Experimentally and theoretically approaches for disperse red 60 dye adsorption on novel quaternary nanocomposites. *Sci. Rep.* 2021, *11*, 10000. https://doi.org/10.1038/s41598-021-89351-9
